# FLIMPA: A Versatile Software for Fluorescence Lifetime Imaging Microscopy Phasor Analysis

**DOI:** 10.1021/acs.analchem.5c00495

**Published:** 2025-05-23

**Authors:** Sofia Kapsiani, Nino F. Läubli, Edward N. Ward, Mona Shehata, Clemens F. Kaminski, Gabriele S. Kaminski Schierle

**Affiliations:** † Department of Chemical Engineering and Biotechnology, 2152 University of Cambridge, Cambridge, CB3 0AS, U.K.; ‡ Analytical Sciences, Bioassay, Biosafety and Impurities, BioPharmaceutical Development, 4625 AstraZeneca, Cambridge CB2 0AA, U.K.

## Abstract

Fluorescence lifetime imaging microscopy (FLIM) is an advanced microscopy technique capable of providing a deeper understanding of the molecular environment of a fluorophore. While FLIM data were traditionally analyzed through the exponential fitting of the fluorophores’ emission decays, the use of phasor plots is increasingly becoming the preferred standard. This is due to their ability to visualize the distribution of fluorescent lifetimes within a sample, offering insights into molecular interactions in the sample without the need for modeling assumptions regarding the exponential decay behavior of the fluorophores. However, so far, most researchers have had to rely on commercial phasor plot software packages which are closed-source and only work with proprietary data formats. In this paper, we introduce FLIMPA, an accessible, open-source, stand-alone software for phasor plot analysis that provides many of the features found in commercial software, and more. FLIMPA is fully developed in Python and offers advanced tools for data analysis and visualization. It enhances FLIM data comparison by integrating phasor points from multiple trials and experimental conditions into a single plot, while also providing the possibility to explore detailed, localized insights within individual samples interactively. We apply FLIMPA to introduce a novel cell-based assay for the quantification of microtubule depolymerization, measured through fluorescence lifetime changes of SiR-tubulin, in response to various concentrations of Nocodazole, a microtubule depolymerizing drug relevant to anticancer treatment.

## Graphical abstract

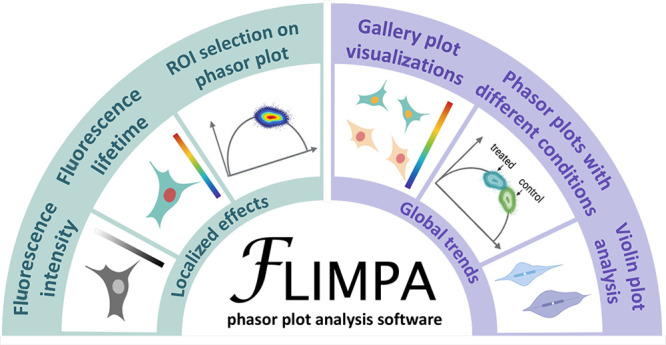

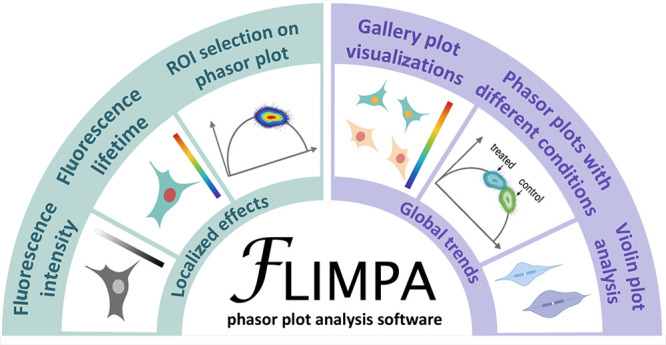

## Introduction

The fluorescence lifetime of a fluorophore is the average time it remains in its excited state before emitting a photon and returning to the ground state.^1^ Variations in fluorescence lifetime can be leveraged to gain insights into the molecular environment of the fluorophores, including changes in temperature, pH, and protein–protein interactions as measured by Förster Resonance Energy Transfer (FRET), among other factors.^2,3^ Fluorescence lifetimes are typically measured by fluorescence lifetime imaging microscopy (FLIM).^4^ FLIM is more robust than conventional fluorescence intensity techniques as it is less susceptible to experimental fluctuations, such as fluctuations in fluorophore concentration, laser intensity, and photobleaching.^3,5^

Over the years, FLIM-based intracellular biosensors have been used to study various biological processes, such as changes in amyloid aggregation state,^6^ calcium concentration,^7^ NAD­(P)H levels,^8^ ATP cleavage,^9^ hydrogen peroxide levels,^10^ redox changes in the endoplasmic reticulum,^11^ and chromatin compaction states.^4^ The predominantly applied FLIM technique is time-correlated single-photon counting (TCSPC)-FLIM, which features a high photon detection efficiency and the smallest temporal resolution.^12,13^ FLIM data can be analyzed either in the time or frequency domain. In the time domain, the fluorescence lifetime parameters can be extracted using curve-fitting techniques through open-source software such as FLIMfit,^14^ FLIMJ,^15^ and FLIMView.^16^ However, these curve-fitting techniques are computationally expensive and demand expert knowledge of the fluorophore’s decay characteristics.^12^ An alternative method to compute the fluorescence lifetime values is via phasor plot analysis,^17^ which uses Fourier transformations to shift the data into the frequency domain.

Unlike traditional fitting, phasor analysis is model-free, computationally efficient, and does not require assumptions on the decay kinetics.^18^ Each image pixel is mapped onto two-dimensional phasor space, thus, enabling the visualization of fluorescence lifetimes distributions within a sample and the detection of subtle differences between populations.^2,13^ However, currently, most software implementations for phasor analysis, such as SPCImage,^19^ Leica LAS X,^20^ FLIM STUDIO,^21^ and VistaVision^18^ are part of commercial microscopy systems, hence, restricting access to their proprietary file formats,^22^ while freely distributed closed-source alternatives like SimFCS^23^ limit transparency and user flexibility. Recently, the open-source Python-based software, FLUTE,^22^ a Napari-Live-FLIM plugin,^24^ Phasor identifier,^25^ and the Python library PhasorPy,^26^ have been published. Unfortunately, while moving in the right direction, these usually offer fewer options for data analysis and usability compared to their commercial counterparts or are not stand-alone applications that can be used without programming expertise.

To address these limitations, we introduce FLIMPA, an open-source, user-friendly, stand-alone software package for phasor plot analysis of TCSPC-FLIM data featuring advanced options for data analysis, visualization, and interpretation, and demonstrate its capabilities through a case study of quantifying microtubule depolymerization upon drug treatment. Microtubules are essential structural components of cells and, therefore, prime targets for anticancer therapies, as disrupting microtubule dynamics inhibits cell division, thus leading to tumor death (Supporting Information, Figure S1).^27,28^ Here, we treated live COS-7 cells with Nocodazole, a microtubule-disrupting agent,^28^ and monitored depolymerization via fluorescence lifetime changes of SiR-tubulin, a small-molecule dye selective for microtubules. This study represents the first application of FLIM in a cell-based assay to measure drug-induced microtubule destabilization.

## Results and Discussion

FLIMPA is designed for phasor plot analysis of raw TCSPC-FLIM data. It is open-source and can be run directly on a Windows computer using the .exe file available on its GitHub repository. FLIMPA serves as a powerful tool for the analysis of bulk FLIM data, offering the capability to visualize phasor plots from multiple samples and different experimental conditions. FLIMPA further supports single-image analysis, allowing users to interactively explore and identify localized effects within individual images, such as the presence of undesired autofluorescence.

An overview of the graphical user interface (GUI) is provided in Figure 1. As shown in Figure 1b, the phasor plots are displayed on the left-hand side of the GUI, while additional visualizations are presented across different tabs on the right-hand side (Figure 1c). An overview of the different tabs is shown in Figure 2, which includes a table listing the phase, modulation, and average fluorescence lifetime values (see Phasor Plot Theory section in Supporting Information) for each sample, visualization of fluorescence lifetime and intensity maps as well as violin plots, to compare fluorescence lifetime distributions between different conditions. All fluorescence lifetime values are reported in nanoseconds (ns).

**Figure 1:**
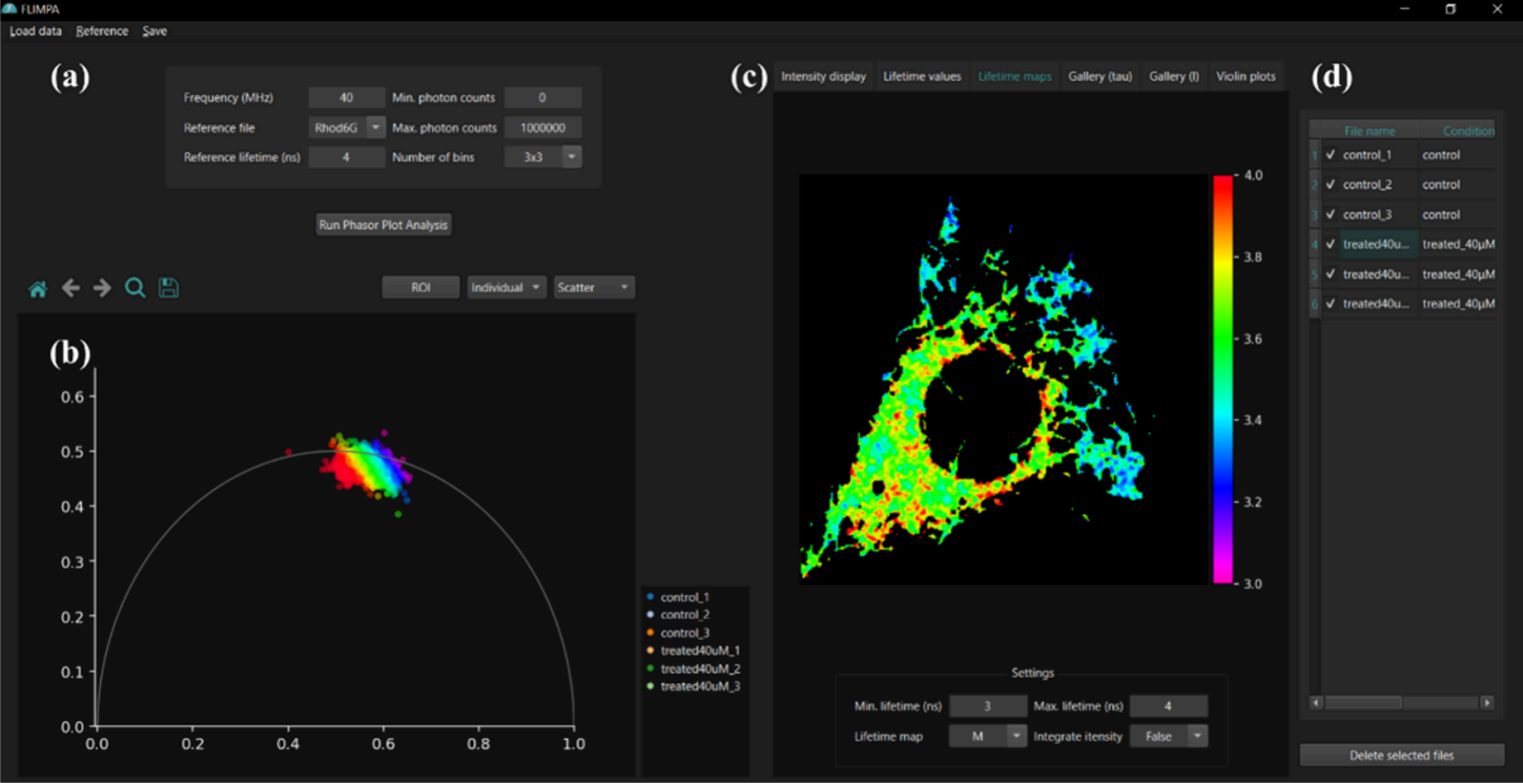
FLIMPA provides a user-friendly interface with (a) an area to set analysis parameters, including laser repetition rate/frequency in Megahertz (MHz), reference file, reference file fluorescence lifetime in nanoseconds (ns), and photon count thresholds; (b) a section for phasor plot visualizations, with the x- and y-axis representing the G-and S-phasor coordinates (for details refer to Phasor Plot Theory section in Supporting Information); (c) different subtabs depicting the postanalysis results, here showing the fluorescence lifetime map; and (d) an overview of the imported file names and their corresponding experimental conditions.

**Figure 2:**
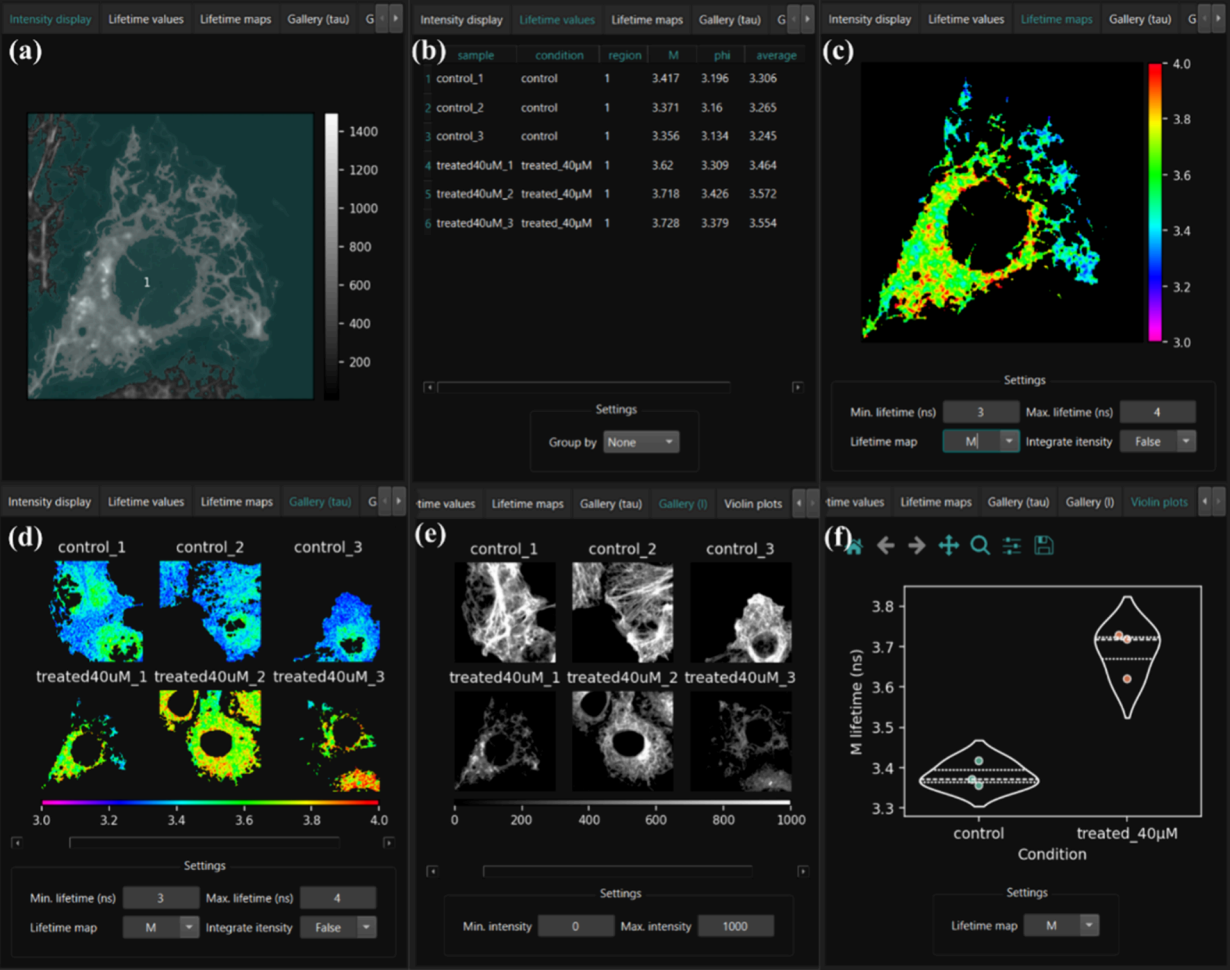
Different tabs of the FLIMPA software permit robust and versatile data analysis. (a) “Intensity display” depicting the input data with intensity masking, if masking has been applied. (b) “Lifetime values” is the output table displaying the mean modulation, phase and average fluorescence lifetime per image. Additionally, the mean fluorescence lifetime, per ROI (if different ROIs have been selected using manual masking) or per experimental condition, can be shown. (c) “Lifetime maps” show the individual images where localized effects can be explored using the ROI selection tool. (d) “Gallery (tau)” is a gallery of the fluorescence lifetime maps for the different samples analyzed. (e) “Gallery (I)” is a gallery of fluorescence intensity maps. (f) “Violin plots” shows the distribution of data points for each treatment group.

Phasor plot analysis requires a reference file of a dye with a known fluorescence lifetime to account for setup variations, with dyes exhibiting monoexponential decay kinetics, such as Rhodamine 6G (fluorescence lifetime of 4 ns in water)^29^ typically being used for system calibration. Further, to ensure accessibility to researchers across various laboratories, FLIMPA supports different file formats, including .sdt (Becker & Hickl), .ptu (PicoQuant), and .tif. Users of .tif files are required to specify the bin width in ns as this format lacks meta-data.

Prior to the analysis, users can mask the background of samples based on the number of photons per pixel by setting minimum and maximum thresholds, as well as by providing manual intensity masks to define regions of interest. Furthermore, to enable comparisons between different experimental conditions or phenotypes, FLIMPA enables users to assign a “condition” to each sample, thus allowing samples of the same set of conditions to be plotted together. Pixel binning is also supported, as commonly applied in FLIMfit,^14^ to aid in the analysis of data with lower signal-to-noise. Finally, for proof-of-principle analysis, we used a specimen of *Convallaria Rhizome* and comparing FLIMPA’s outputs to FLIMfit^14^ highlighted comparable results (Supporting Information, Figure S2).

### FLIMPA enables the investigation of localized fluorescence lifetime changes within individual images

FLIMPA allows users to interactively explore variations within individual images using the “Lifetime Maps” tab, where both the fluorescence lifetime image and the corresponding phasor points are color-coded based on each pixel’s fluorescence lifetime value. The ROI selection tool links the selected phasor plot points to their corresponding pixels on the fluorescence lifetime image, thus enabling users to identify outliers and examine their location, cellular structure, or treatment response. As illustrated in Figure 3, several pixels of lower fluorescence lifetimes, colored in pink, are shown as separated from the main phasor cluster. Specifically, Figure 3b and Figure 3c present the fluorescence lifetime and intensity images of microtubules labeled with SiR-tubulin and treated with Nocodazole. Remapping the individual pink dots observed through the phasor plot-based segmentation to the lifetime images suggests that these outliers correspond to aggregated Sir-tubulin monomers that detached from cells. Indeed, Pineda et al. (2018)^30^ also reported that SiR-tubulin is prone to aggregation which leads to increased self-quenching and a decrease in fluorescence lifetime. Additionally, the intensity image shown in Figure 3c verifies that these regions do not correspond to the microtubule organizing center (MTOC) which, due to its tight arrangement, could also display a lower fluorescence lifetime.

**Figure 3:**
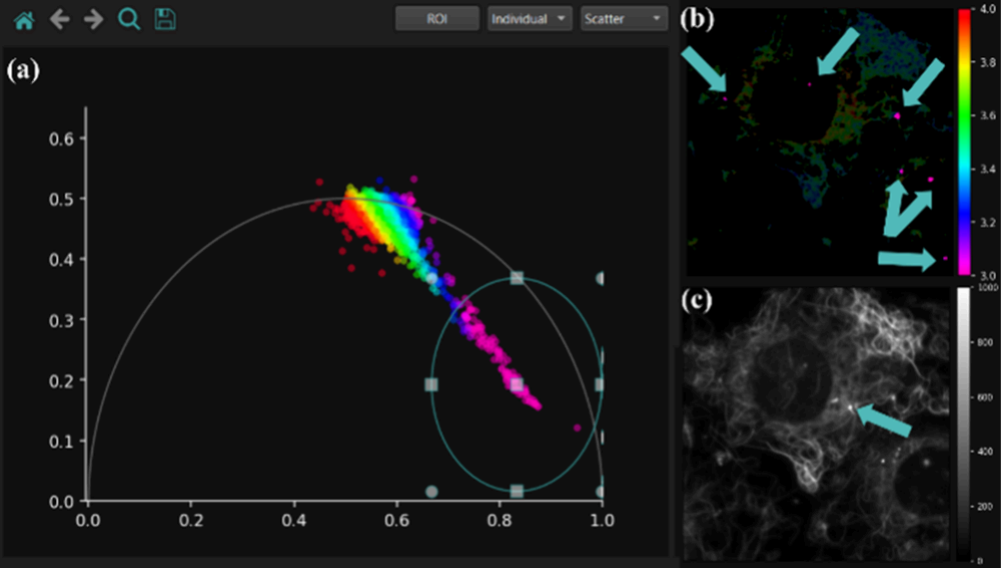
ROI selection tool can be used to interactively explore different regions within the sample. (a) Phasor points with lower lifetimes compared to the main phasor cluster, selected with the ROI tool. (b) The fluorescence lifetime map highlights pixels corresponding to the ROI-selected phasor in pink. These regions indicated by blue arrows likely represent SiR-tubulin aggregates and are manually excluded from the data analysis. (c) The intensity image with an arrow indicating the microtubule organizing center (MTOC) confirms that the phasor points with lower lifetime do not represent the cell’s MTOC.

### FLIMPA is a powerful tool for studying global trends across different experimental conditions

FLIMPA’s “Gallery (tau)” tab enables users to combine phasor points assigned with the same experimental condition into single clouds, thus allowing comparisons across different phenotypes or treatment conditions. Here, FLIMPA has been used to analyze how different concentrations of Nocodazole, specifically 0 μM (control), 1 μM, 10 μM, and 40 μM, affect the fluorescence lifetime of SiR-tubulin and microtubule stability. As also seen above, SiR-tubulin, as a rhodamine-based dye, undergoes self-quenching when molecules are in close proximity which leads to a reduction in its fluorescence lifetime.^31^ Intact microtubules therefore promote self-quenching due to the proximity of dye molecules when attached to adjacent tubulin subunits. On the other hand, Nocodazole-induced depolymerization disperses the tubulin subunits, thus reducing self-quenching and increasing the SiR-tubulin fluorescence lifetime.

Figure 4a compares phasor clusters between control and 10 μM Nocodazole-treated COS-7 cells. Treatment with Nocodazole shifted the distribution of fluorescence lifetimes toward the left-hand side of the phasor plot’s universal circle, indicating a higher degree of microtubule depolymerization. In addition to the contour map displayed in Figure 4a, FLIMPA offers scatter plots and density-sensitive histogram visualization options (Supporting Information, Figure S3). The phasor cloud comparisons for all tested Nocodazole concentrations are shown in Supporting Information, Figure S4.

**Figure 4:**
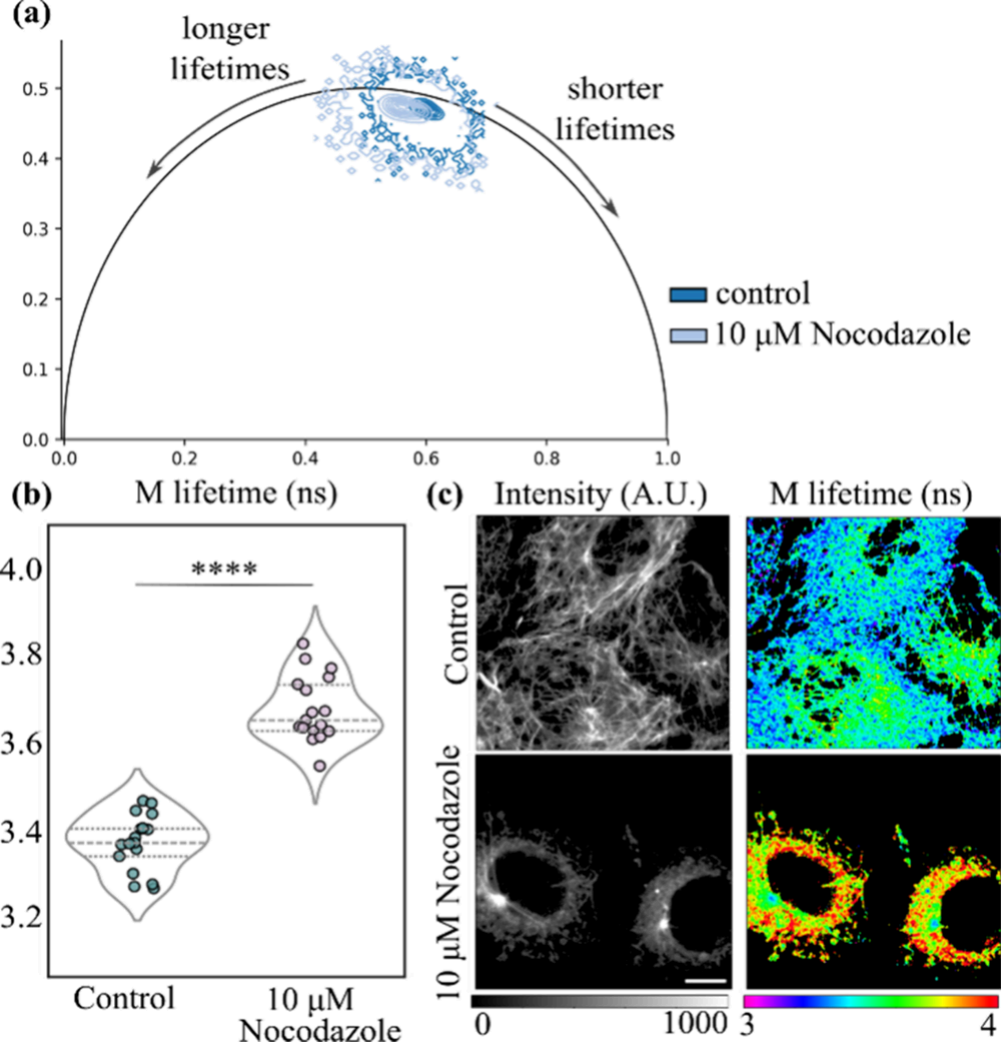
Data analysis performed on FLIMPA reveals that 10 μM Nocodazole significantly destabilizes microtubules. (a) Phasor plot exported from FLIMPA displaying contour maps for control (darker blue) versus 10 μM Nocodazole (light blue). (b) Violin plots of SiR-tubulin modulation (M) lifetime in COS-7 cells treated with 0 μM (control) and 10 μM Nocodazole, where the mean modulation (M) lifetimes are 3.39 and 3.69 ns, respectively. The lines within the plots indicate the interquartile range and median. Statistical significance is calculated using the Mann–Whitney test, where **** indicates a p-value <0.0001. Statistical analysis was performed in Python using fluorescence lifetime values exported from FLIMPA. *n* = 17 images per condition from four independent repeats. (c) Example intensity and M lifetime maps of live COS-7 microtubules treated with 0 μM (control) and 10 μM Nocodazole generated using FLIMPA. The scale bar is 10 μm. The figure was edited in Inkscape to indicate the shift toward longer and shorter lifetimes on the phasor plot and highlight significance levels on the violin plots.

For statistical analysis, fluorescence lifetime values were exported as .csv files and analyzed using the Mann–Whitney test in Python. Here, we focused on the modulation lifetime for the analysis, as it exhibited a higher sensitivity to Nocodazole treatment. The analysis revealed a statistically significant increase in SiR-tubulin modulation lifetimes after treatment with 10 μM Nocodazole compared to control conditions (Figure 4b). A one-way ANOVA with Tukey’s multiple comparisons test performed across all tested Nocodazole concentrations () demonstrated that 10 μM Nocodazole is enough to completely destabilize COS-7 microtubules, with no further significant increase in modulation lifetimes observed with the 40 μM Nocodazole treatment.

Finally, to confirm that Nocodazole alone does not directly alter SiR-tubulin fluorescence lifetimes, the dye was imaged in solution on a coverslip, showing no change in fluorescence lifetime upon the addition of Nocodazole (). However, similar to Figure 3, SiR-tubulin aggregation was observed, thus providing further evidence that the previously described phasor points of lower fluorescence lifetime values reflect dye clustering rather than cellular structures.

## Conclusion

We have introduced FLIMPA, an easy-to-use, open-source, and stand-alone software package for phasor plot analysis, developed in Python. FLIMPA allows researchers to investigate local variations within individual FLIM images as well as to examine fluorescence lifetime differences across multiple samples, extending the capabilities of other open-source phasor plot applications like FLUTE.^22^ Unlike the Napari-Live-FLIM plug-in,^24^ FLIMPA is a stand-alone application that is straightforward to execute without requiring any coding expertise. Furthermore, in contrast to commercial alternatives that typically restrict analysis to proprietary file formats, FLIMPA supports multiple file types, ensuring broader accessibility.

Utilizing FLIMPA, we introduced a novel FLIM-based assay for quantifying microtubule network disruption upon Nocodazole treatment by measuring the fluorescence lifetime changes of SiR-tubulin. In future work, we will extend the assay to other depolymerizing agents, such as colchicine and vinblastine, and thus establish FLIMPA as a useful tool for studying drug-induced structural changes and identifying drug candidates relevant to cancer research. Finally, due to its open-source nature, FLIMPA can be easily adapted to analyze data from other imaging modalities, such as hyperspectral imaging, with tools like PhasorPy^26^ serving as a baseline for incorporating hyperspectral data analysis.

## Supplementary Material





## Data Availability

The executable file, Python code and sample data can be found on FLIMPA’s GitHub repository (https://github.com/SofiaKapsiani/FLIMPA).

## Notes

Methods, phasor plot theory, Python system requirements, cell culture, FLIM-TCSPC setup and additional figures (PDF)
User Manual (PDF)
