# Supplementary material for: FLIMPA: A Versatile Software for Fluorescence Lifetime Imaging Microscopy Phasor Analysis
Source: Anal Chem. 2025 May 23;97(22):11382–7. doi: 10.1021/acs.analchem.5c00495 (PMC12163867; doi:10.1021/acs.analchem.5c00495)
Supplement: Supplementary file 1 [file ac5c00495_si_001.pdf]

# Supporting Information

# FLIMPA: A Versatile Software for Fluorescence Lifetime Imaging Microscopy Phasor Analysis

Sofia Kapsiani[1], Nino F. Läubli[1], Edward N. Ward[1], Mona Shehata[2], Clemens F. Kaminski[1], Gabriele S. Kaminski Schierle[1]

[1]Department of Chemical Engineering and Biotechnology, University of Cambridge, Cambridge, CB3 0AS, UK
[2]Analytical Sciences, Bioassay, Biosafety and Impurities, BioPharmaceutical Development, AstraZeneca, Cambridge, UK

* Corresponding author: gsk20@cam.ac.uk

**Contents**

**Methods**

Phasor Plot Theory ........ 2

Implementation ........ 3

Manual mask generation ........ 3

System requirements ........ 4

Cell culture ........ 4

In-house TCSPC set-up ........ 4

References ........ 12

**Supplementary Figures**

**Figure S1.** Overview of case study on quantifying microtubule depolymerisation upon drug exposure ........ 5

**Figure S2.** Overview of the FLIMPA GUI displaying the phasor plot analysis of a Convallaria Rhizome sample ........ 6

**Figure S3.** Phasor clouds visualisation options provided by FLIMPA ........ 7

**Figure S4.** FLIMPA reveals that increasing concentrations of Nocodazole significantly destabilise microtubules ........ 8

**Figure S5.** Data analysis performed on exported FLIMPA code reveals that 10 $\mu$M Nocodazole already completely destabilises microtubules ........ 9

**Figure S6.** Studying the effect of Nocodazole on SiR-tubulin fluorescence lifetime imaged on a coverslip ........ 10

**Figure S7.** SiR-tubulin molecule aggregation imaged on a coverslip ........ 11

## Methods

### Phasor Plot Theory

In time-domain FLIM, the output data are three-dimensional, where the $x$, $y$- dimensions correspond to the spatial information while the z-dimension is the change of fluorescence intensity with time, $I(t)$, given by Eq. (1):[1]

$$I(t) = \sum_{n} \alpha_n e^{-\frac{t}{\tau_n}} \tag{1}$$

Where $t$ is time, $\alpha_n$ the fractional contribution of each lifetime component $\tau_n$ and $n$ is the number of components.

The fluorescence decay curve can be transformed to the frequency domain using the Fourier transform as expressed by Eq. (2):[2]

$$\hat{I}(\omega) = \int_0^{\infty} I(t) e^{-ik\omega t}\, dt \tag{2}$$

Where $\omega$ is the angular frequency, $I$ is intensity, $i$ is the imaginary component, and $k$ is the harmonic number[2]. The angular frequency is derived via the repetition rate of the laser $f$ as $\omega = 2\pi f$. In this work, the phasor plot analysis is performed using the first harmonic, therefore $k$ is set to 1.

The phasor coordinates, $g$ — and $s$ — of the real (cosine) and imaginary (sine) parts of the Fourier Transform for a given pixel $(x, y)$ are calculated as follows by Eq. (3) and Eq. (4), respectively:[3]

$$g_{x,y}(\omega) = \frac{\int_0^{\infty} I_{x,y}(t) \cos(\omega t)\, dt}{\int_0^{\infty} I_{x,y}(t)\, dt} \tag{3}$$

$$s_{x,y}(\omega) = \frac{\int_0^{\infty} I_{x,y}(t) \sin(\omega t)\, dt}{\int_0^{\infty} I_{x,y}(t)\, dt} \tag{4}$$

In frequency domain FLIM, the $g$- and $s$- coordinates at each pixel $(x, y)$ can be calculated from the modulation ratio $m_{(x,y)}$ and phase delay $\phi_{(x,y)}$ using Eq. (5) and Eq. (6):[3]

$$g_{x,y}(\omega) = m_{x,y} \cos(\phi_{x,y}) \tag{5}$$

$$s_{x,y}(\omega) = m_{x,y} \sin(\phi_{x,y}) \tag{6}$$

By plotting $g$- and $s$- coordinates, each pixel is represented by a point within the phasor plot.[4] The semi-circle is known as the universal circle, where points corresponding to shorter lifetimes are placed closer to the right-hand side of the universal circle $(g, s = 1{,}0)$ while points of longer lifetimes are closer to the origin of the circle $(g, s = 0{,}0)$, as shown in Methods Figure ***1***. Moreover, phasor points of mono-exponential decays lie on the universal circle while points calculated from multi-exponential decays fall within the circle.[3]

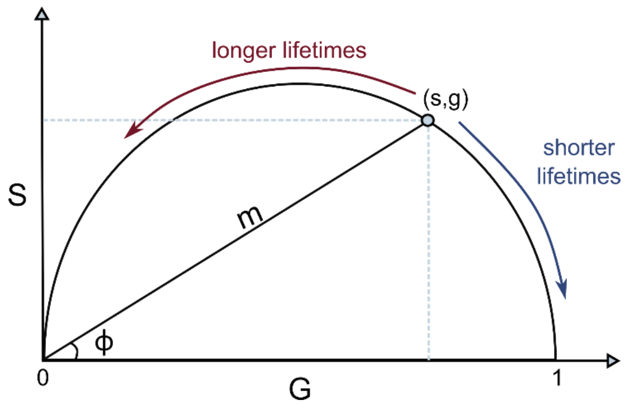


**Methods Figure 1.** Schematic of a phasor plot, where the phase angle is denoted by $\varphi$ and the modulation $m$ is shown as a vector. The figure was recreated in Inkspace from Sun *et al.* 2014.[3]

Using trigonometric functions, the modulation and phase can be derived from the $g$- and $s$- coordinates of a given point by Eq. (7) and Eq. (8):[5]

$$m = \sqrt{g^2 + s^2} \tag{7}$$

$$\phi = \arctan\left(\frac{s}{g}\right) \tag{8}$$

The fluorescence lifetime corresponding to the modulation and phase, given by Eq. (9) and Eq. (10)[5], respectively, are referred to as "modulation lifetime" and "phase lifetime".

$$\tau_m = \frac{1}{\omega}\sqrt{\frac{1}{m^2} - 1} \tag{9}$$

$$\tau_\phi = \frac{1}{\omega}\tan(\phi) \tag{10}$$

For mono-exponential species the phase and modulation lifetimes are equal.[6] Additionally, an average of the phase and modulation lifetime can be taken, which is referred to as the "average lifetime" in FLIMPA. To account for instrumental factors, such as differences in the detector and optics, a reference file with a known lifetime is used for calibration. Commonly, Rhodamine 6G which has a known lifetime of 4 ns is used as the reference sample. Other unquenched dyes can also be used, with the reference correction serving a similar purpose as the Instrumental Response Function (IRF) deconvolution in curve-fitting methods.

## Implementation

FLIMPA was developed using Python (version 3.11.7), with the phasor plot analysis predominantly performed using the NumPy (version 2.0.0)[7] and SciPy (version 1.14.0)[8] libraries. The frontend of the application was built using PySide6 (version 6.7.2), while plotting and visualisation were achieved using Matplotlib[9] (version 3.9.1), Seaborn (version 0.13.2),[10] and Pandas (version 2.2.2).[11] The complete list of libraries used for building FLIMPA is available in the requirements.txt file on GitHub (https://github.com/SofiaKapsiani/FLIMPA). Statistical analysis of the exported data was conducted using the Pingouin library (version 0.5.4).[12]

## Manual mask generation

To remove areas of SiR-tubulin molecule clustering, manual masks of the COS-7 microtubules were created. This involved first segmenting the background of the microtubules in Python (version 3.11.7) by setting a threshold of 200 photon counts per pixel, followed by importing the masks generated using FLIMfit[13] and manually removing the areas that corresponded to the dye aggregation.

### System requirements

FLIMPA can be executed on a Windows computer using the .exe file provided on its GitHub (https://github.com/SofiaKapsiani/FLIMPA) repository. The repository also includes FLIMPA's backend and frontend Python code. The sample .sdt files are provided so that the GUI can be easily tested. The user manual for FLIMPA can be found as a separate file in Supporting Information. The processing speed of FLIMPA was evaluated using 72 Becker & Hickl .sdt files with dimensions of 256x256x256, where each image took an average of 1.4 seconds to process without pixel binning, and 1.8 seconds with 3x3 pixel binning applied. These results were obtained using a system running Windows 10, equipped with an Intel(R) Core(TM) i7-9750H CPU @ 2.60GHz.

### Cell culture

COS-7 cells were acquired from the American Type Culture Collection (ATCC, USA) and cultured at 37 °C with a 5% $CO_2$ supply. Cells were grown in Dulbecco's modified Eagle's medium (DMEM, Thermo Fisher Scientific, USA) supplemented with 10% fetal bovine serum (FBS, Thermo Fisher Scientific), 1% penicillin-streptomycin and 1% of GlutaMAX™ (Thermo Fisher Scientific). 10K cells were seeded in each chamber of an 8-chamber glass well plate (IBIDI GmbH, Germany) with 200 µL of cell media and incubated at 37 °C with 5% $CO_2$ for 24 hours. This was followed by overnight staining with 1 $\mu$M SiR-tubulin (Spirochrome, Switzerland) and 10 $\mu$M Verapamil (Spirochrome) in cell media. Verapamil is an efflux pump inhibitor and was added to the staining solution to prevent the cells from expelling the fluorescent probe. The following day, cells were washed twice with phosphate-buffered saline (PBS, Thermo Fisher Scientific) and 200 $\mu$L of fresh cell media with 10 $\mu$M Verapamil was added. After media replacement, the cells were treated with 1 $\mu$M, 10 $\mu$M, or 40 $\mu$M of Nocodazole for 30 minutes. Imaging occurred immediately following treatments, without removal of the Nocodazole, during which an on-stage incubator system (OKOLab, Italy) was used to maintain the cells at 37 °C and 5% $CO_2$.

### In-house TCSPC set-up

The in-house TCSPC module is based on a confocal microscope. The samples were illuminated with a pulsed laser (Fianium Whitelase, Denmark) at 40 MHz frequency[14]. Imaging was performed using an Olympus IX83 microscope system and a 60x oil objective with a 1.40 numerical aperture (Olympus, Japan). The fluorescent photons were captured using a Becker & Hickl GmbH PMC-150 photon multiplier tube (PMT) and SPC-830 Photon Counting Electronics[14]. Photons are accumulated over 10 cycles with a duration of 12 seconds each, with output data having the dimensions of 256x256x256 (t, x, y). The excitation and emission filters were centred at 635 nm and 700 nm (FF02-632/22-25, Semrock Inc, USA and ET700/75m, Chroma, USA)[14]. Rhodamine 6G (at a concentration of 250 $\mu$M in $H_2O$) was used as the reference sample and measured using excitation and emission filters centred at 510-20 nm and 542-27 nm (FF03-510/20-25 and FF01-542/27-25, Semrock Inc), respectively. The image of the *Convallaria Rhizome* sample (shown in Figure S2) was acquired using the 510-20 nm and 542-27 nm filters and a 40x oil Olympus objective with a 1.30 numerical aperture. To prevent photon pile-up, the photon counts at the detector were kept below 1% of the instrumental frequency.

## Supplementary Figures

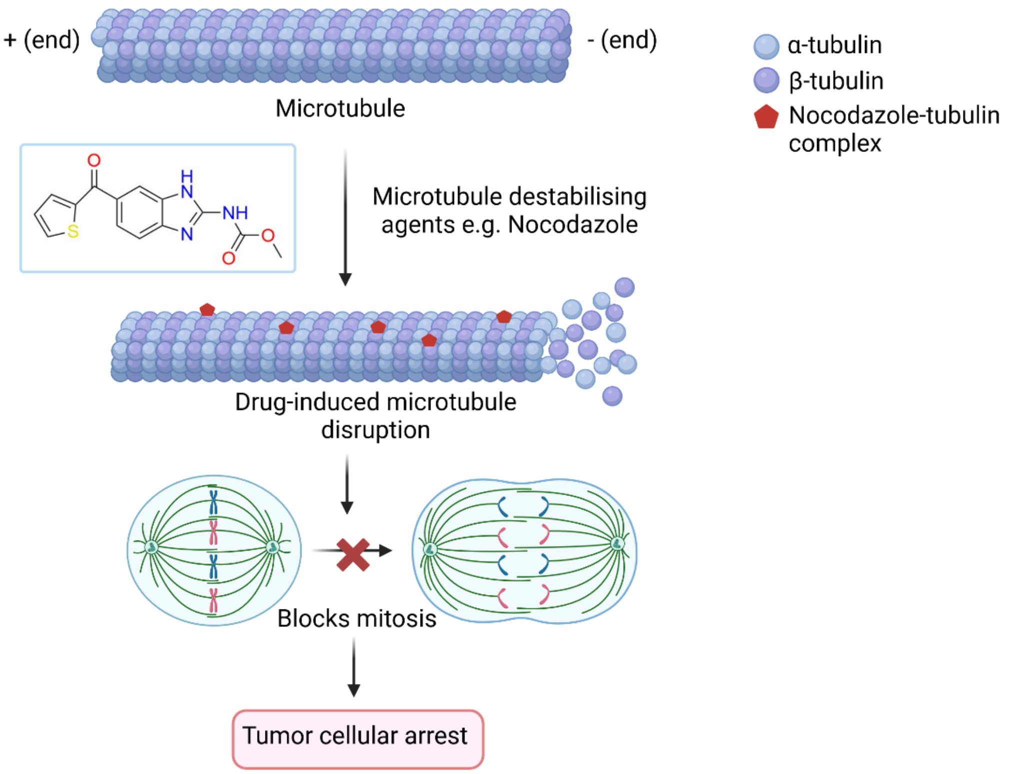


**Figure S1.** Overview of case study on quantifying microtubule depolymerisation upon drug exposure. Microtubule destabilising agents, such as Nocodazole, bind to the microtubules and induce the disassembly of the tubulin subunits leading to mitotic arrest and cell death. A FLIM-based assay was used to quantify the drug-induced microtubule depolymerisation by measuring the fluorescence lifetime of SiR-tubulin, i.e., a small dye molecule that selectively binds to the β-subunits of microtubules. The schematic was recreated from Gupta et al. (2019)[15] using BioRender with the Nocodazole structure designed in ChemDraw.

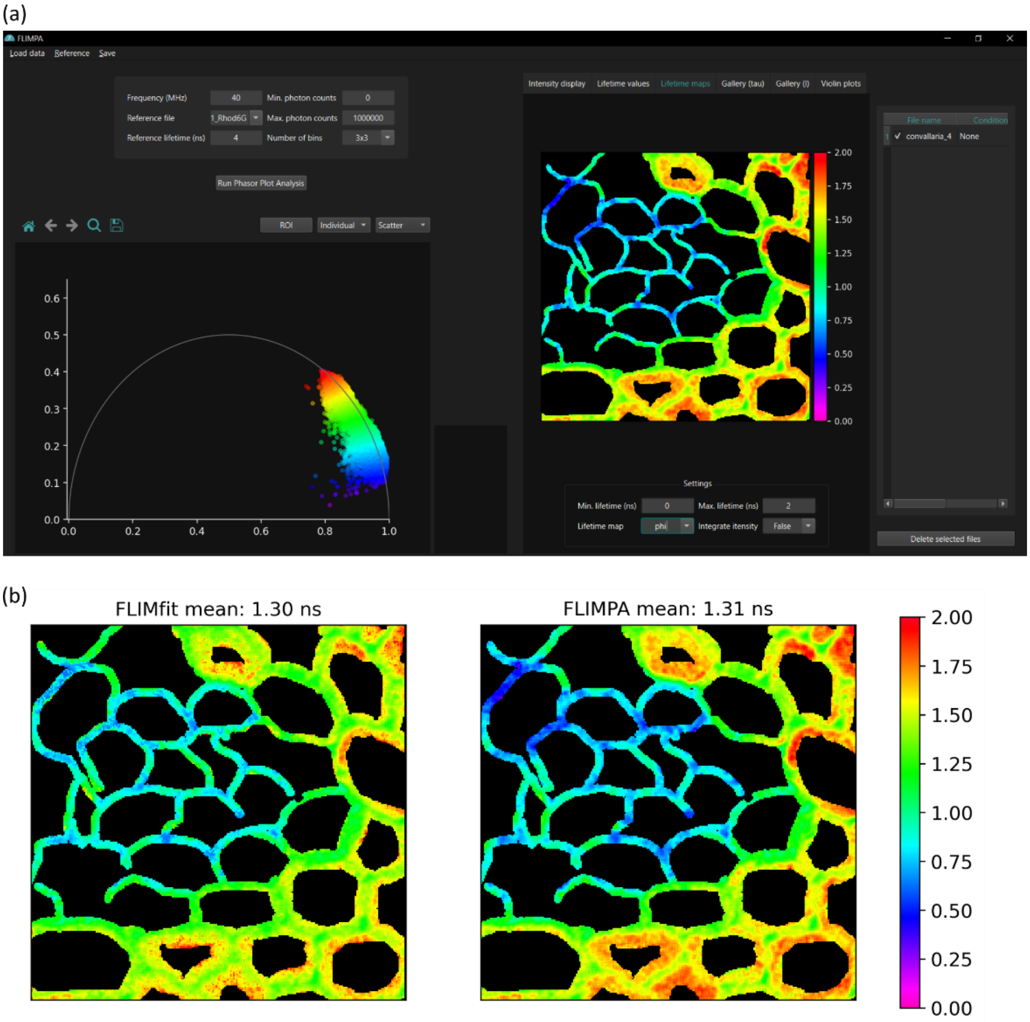


**Figure S2.** (a) Overview of the FLIMPA GUI displaying the phasor plot analysis of a *Convallaria Rhizome* sample. (b) Comparison between the bi-exponential fluorescence lifetime image of *Convallaria Rhizome* from FLIMfit[13] (left) and the phase lifetime map generated by FLIMPA (right). The mean bi-exponential fluorescence lifetime and mean phase lifetime are indicated in the image subtitles. Both images were plotted in Python, with the colour bar representing time in nanoseconds.

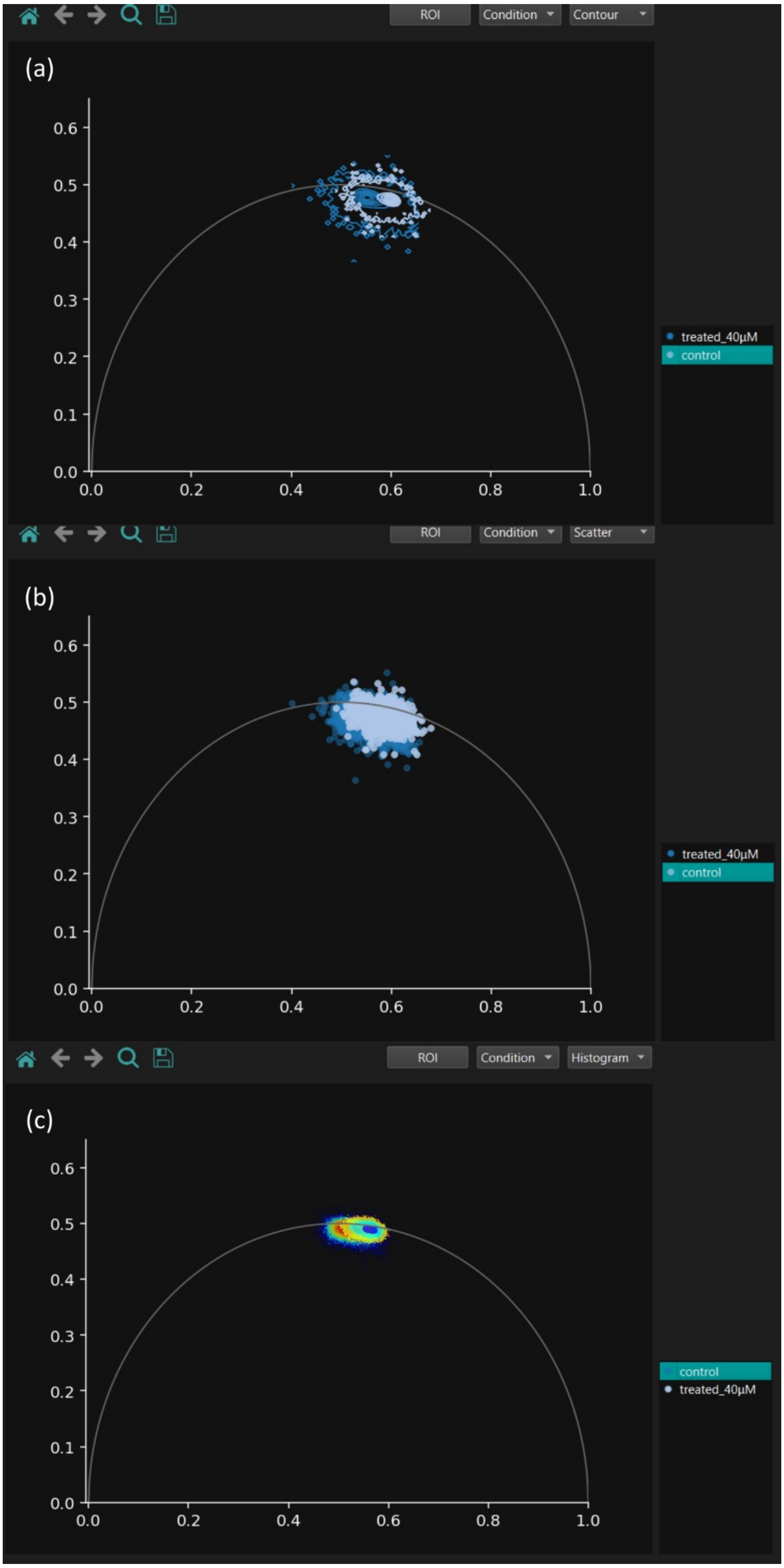


**Figure S3.** Phasor clouds visualisation options provided by FLIMPA using (a) contour maps, (b) scatter plots, and (c) density-sensitive histograms.

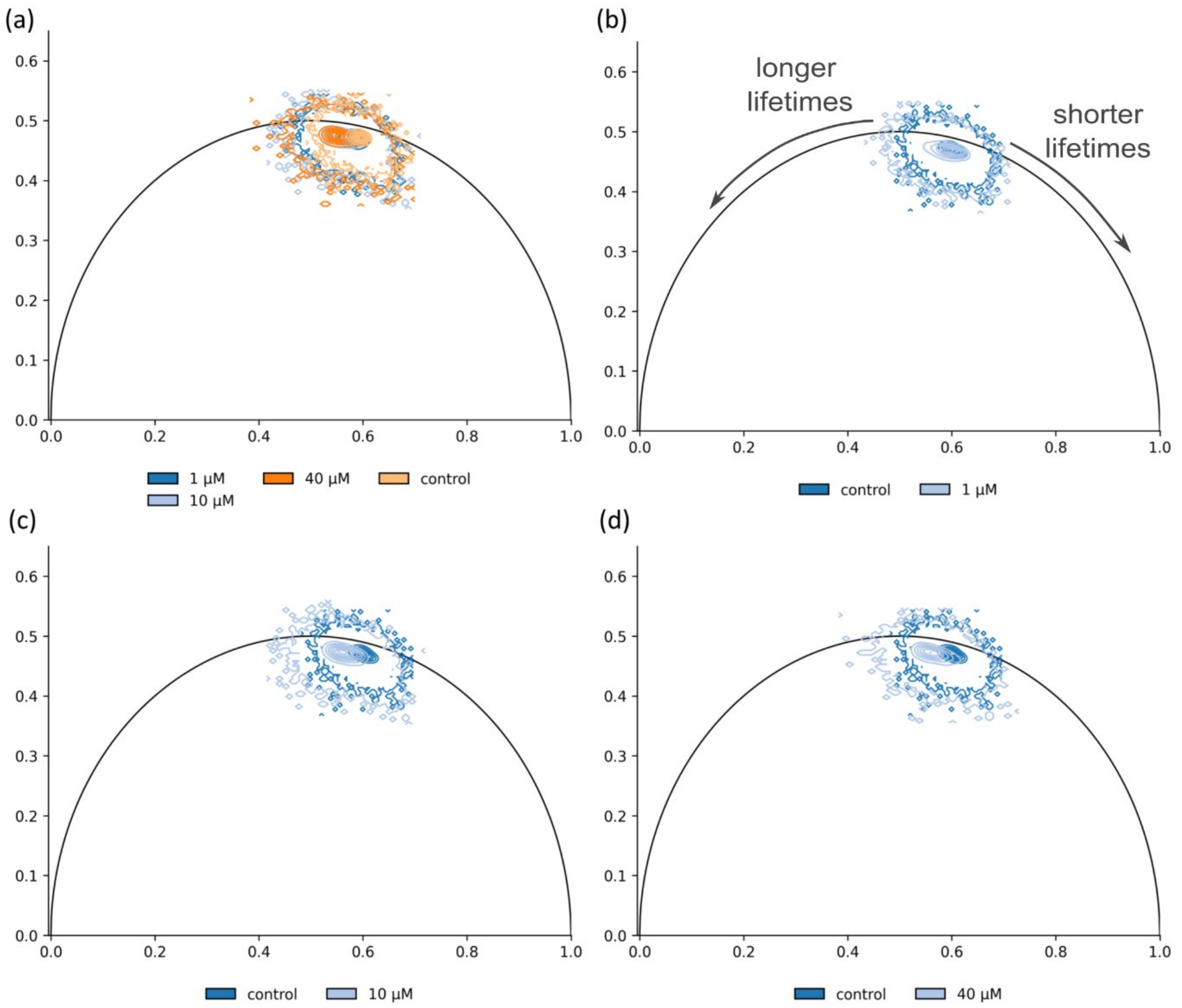


**Figure S4.** FLIMPA reveals that increasing concentrations of Nocodazole significantly destabilise microtubules. Phasor plots exported from FLIMPA displaying contour maps for (a) control (light orange), 1 $\mu$M Nocodazole (darker blue), 10 $\mu$M Nocodazole (light blue), and 40 $\mu$M Nocodazole (darker orange); (b) control (darker blue) versus 1 $\mu$M Nocodazole (light blue); (c) control (darker blue) versus 10 $\mu$M Nocodazole (light blue); and (d) control (darker blue) versus 40 $\mu$M Nocodazole (light blue). The figure was edited in Inkscape to indicate the shift towards longer and shorter lifetimes.

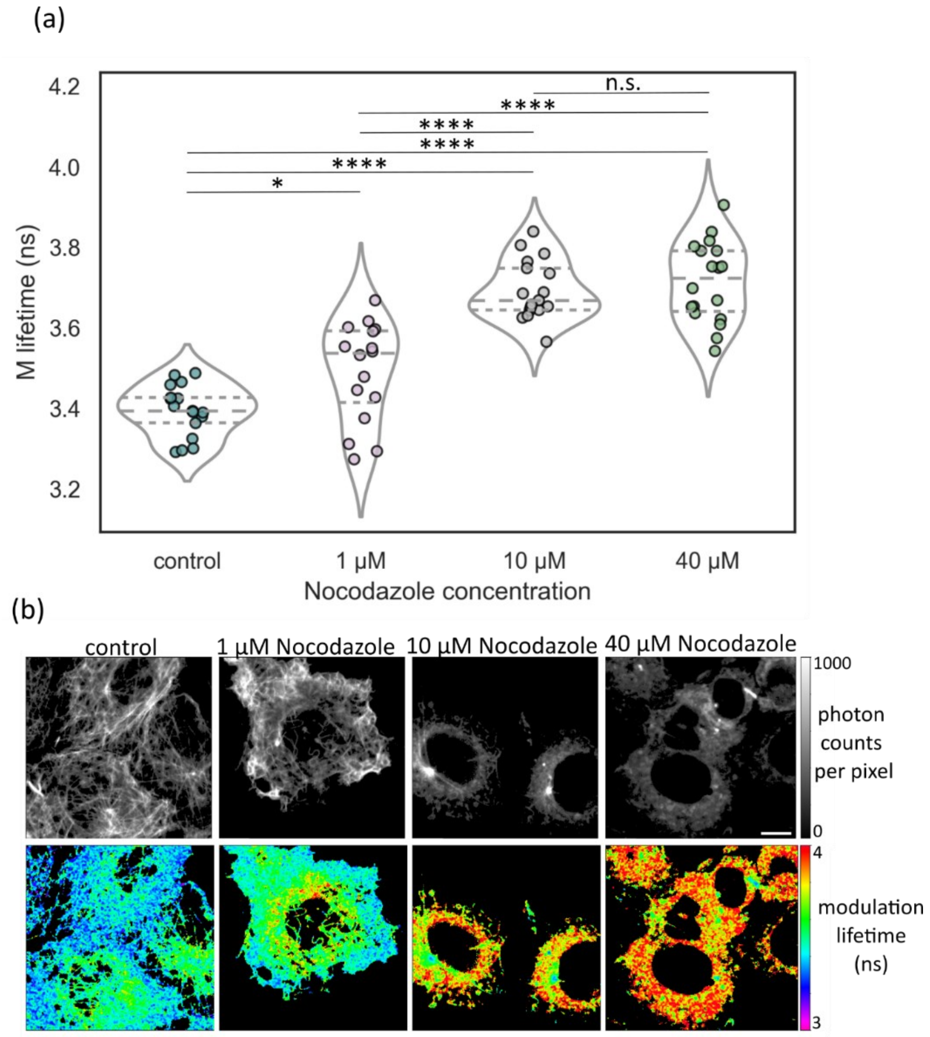


**Figure S5.** Data analysis performed on exported FLIMPA code reveals that 10 $\mu$M Nocodazole already completely destabilises microtubules. (a) Violin plots of SiR-tubulin modulation lifetime in COS-7 cells treated with 0 $\mu$M (control), 1 $\mu$M, 10 $\mu$M, and 40 $\mu$M Nocodazole. The plots were generated in Python using fluorescence lifetime values exported from FLIMPA. The lines within the plots indicate the interquartile range and median. The mean SiR-tubulin modulation lifetime is 3.39 ns, 3.49 ns, 3.69 ns and 3.71 ns for the control, 1 $\mu$M, 10 $\mu$M, and 40 $\mu$M Nocodazole, respectively. The statistical analysis was performed using one-way ANOVA with Tukey multiple comparisons, where * indicates a p-value < 0.05, **** indicates a p-value < 0.0001, and n.s. denotes non-significant results. The plot was edited in Inkscape to highlight significance levels. Data were collected from four independent repeats. (b) Example intensity and modulation lifetime maps of COS-7 microtubules treated with different Nocodazole concentrations generated using FLIMPA. Scale bar is 10 $\mu$m.

(a)

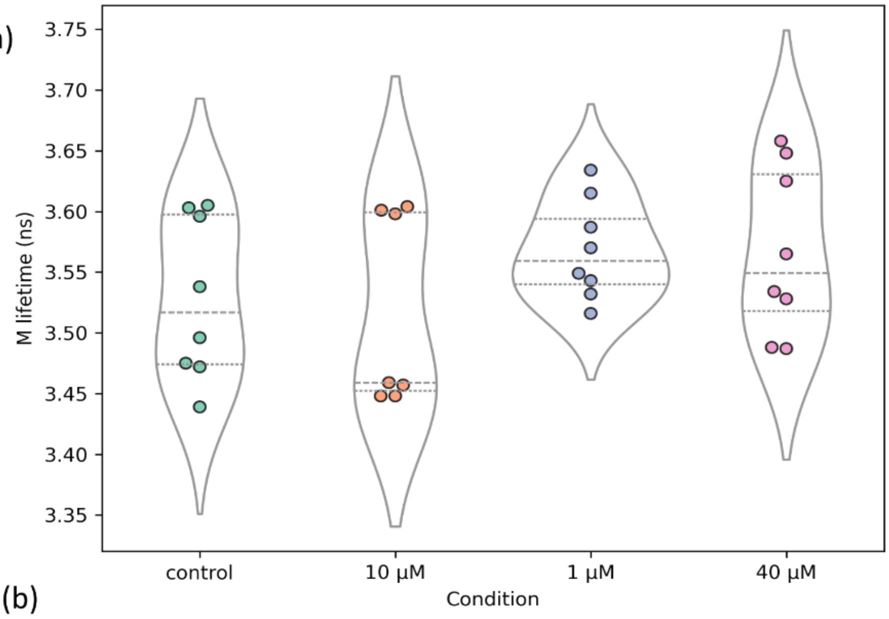


(b)

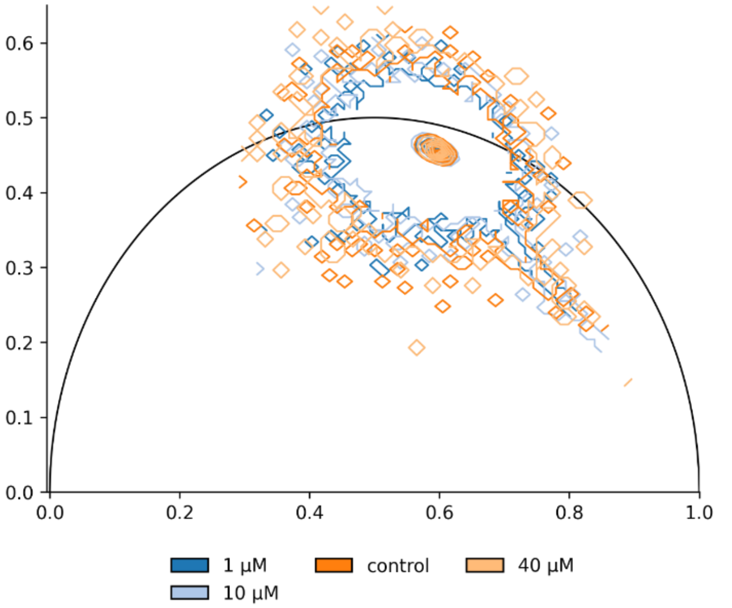


(c)

| Condition | M lifetime (ns) |
|---|---|
| control | 3.53 |
| 1 µM | 3.57 |
| 10 µM | 3.52 |
| 40 µM | 3.57 |

**Figure S6.** Studying the effect of Nocodazole on SiR-tubulin fluorescence lifetime imaged on a coverslip. (a) Violin plots showing the effect of different concentrations of Nocodazole on SiR-tubulin modulation lifetime; (b) phasor plot exported from FLIMPA for control (darker orange), 1 $\mu$M Nocodazole (darker blue), 10 $\mu$M Nocodazole (light blue), and 40 $\mu$M Nocodazole (light orange); (c) table of modulation lifetimes in nanoseconds per Nocodazole concentration.

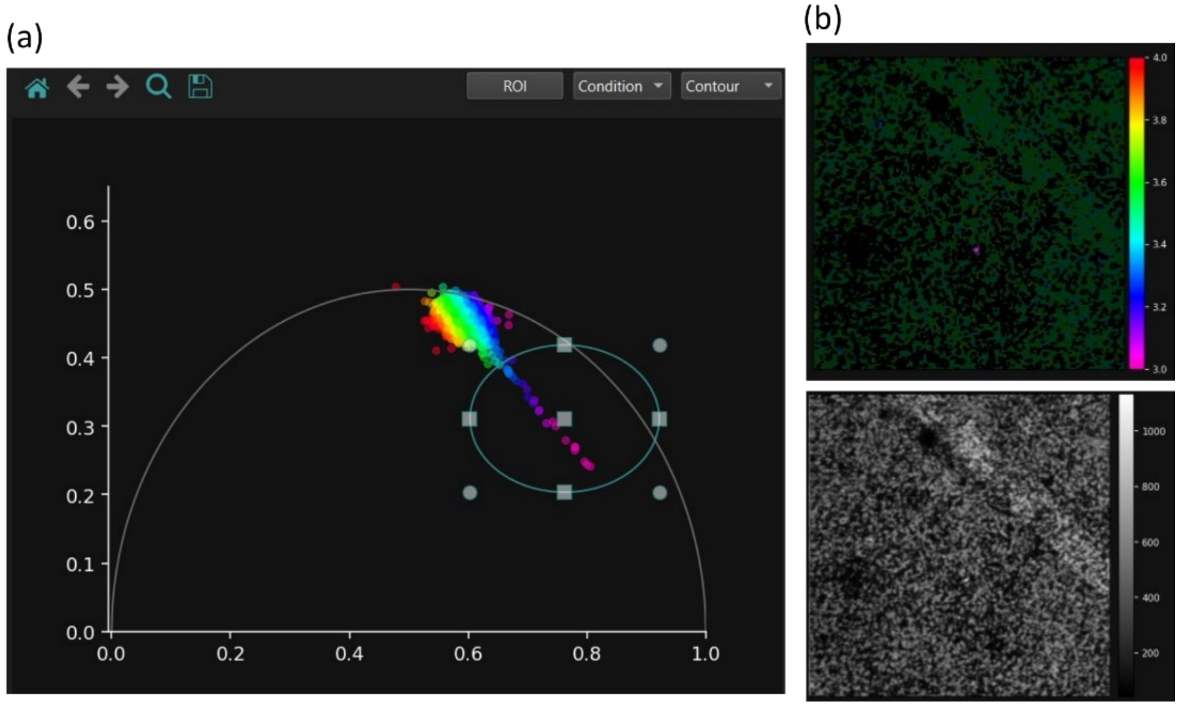


**Figure S7.** SiR-tubulin molecule aggregation. (a) Phasor plot from a sample of SiR-tubulin imaged on a coverslip where the phasor points with lower fluorescence lifetime, selected using the ROI tool, correspond to dye aggregation. (b) Corresponding modulation fluorescence lifetime map highlighting the pixels associated with dye clustering (top) and intensity map (bottom).

**References**


(1) Datta, R.; Heaster, T. M.; Sharick, J. T.; Gillette, A. A.; Skala, M. C. Fluorescence Lifetime Imaging Microscopy: Fundamentals and Advances in Instrumentation, Analysis, and Applications. *J Biomed Opt* **2020**, *25* (7), 71203.

(2) Lakner, P. H.; Monaghan, M. G.; Möller, Y.; Olayioye, M. A.; Schenke-Layland, K. Applying Phasor Approach Analysis of Multiphoton FLIM Measurements to Probe the Metabolic Activity of Three-Dimensional in Vitro Cell Culture Models. *Sci Rep* **2017**, *7* (1), 42730.

(3) Sun, Y.; Liao, S.-C. The Ultimate Phasor Plot and Beyond. *ISS Inc* **2014**.

(4) Adhikari, M.; Houhou, R.; Hniopek, J.; Bocklitz, T. Review of Fluorescence Lifetime Imaging Microscopy (FLIM) Data Analysis Using Machine Learning. *Journal of Experimental and Theoretical Analyses* **2023**, *1* (1), 44–63.

(5) Gottlieb, D.; Asadipour, B.; Kostina, P.; Ung, T. P. L.; Stringari, C. FLUTE: A Python GUI for Interactive Phasor Analysis of FLIM Data. *Biological Imaging* **2023**, *3*, e21.

(6) Schwarz, J.; Wohlschläger, M.; Leiter, N.; Auer, V.; Risse, M.; Versen, M. Frequency Domain Fluorescence Lifetime Imaging Microscopy (FD-FLIM) Analysis of Quercus Robur Samples for Origin Differentiation Purposes. In *Fourier Transform Spectroscopy*; 2023; pp JTu4A–10.

(7) Harris, C. R.; Millman, K. J.; der Walt, S. J. van; Gommers, R.; Virtanen, P.; David Cournapeau; Wieser, E.; Taylor, J.; Sebastian Berg; Smith, N. J.; Kern, R.; Hoyer, M. P. and S.; van Kerkwijk, M. H.; Matthew Brett; Haldane, A.; del Río, J. F.; Wiebe, M.; Peterson, P.; Pierre Gérard-Marchant; Sheppard, K.; Reddy, T.; Weckesser, W.; Abbasi, H.; Gohlke, C.; Oliphant, T. E. Array Programming with NumPy. *Nature* **2020**, *585* (7825), 357–362. https://doi.org/10.1038/s41586-020-2649-2.

(8) Virtanen, P.; Gommers, R.; Oliphant, T. E.; Haberland, M.; Reddy, T.; Cournapeau, D.; Burovski, E.; Peterson, P.; Weckesser, W.; Bright, J.; van der Walt, S. J.; Brett, M.; Wilson, J.; Millman, K. J.; Mayorov, N.; Nelson, A. R. J.; Jones, E.; Kern, R.; Larson, E.; Carey, C. J.; Polat, \.Ilhan; Feng, Y.; Moore, E. W.; VanderPlas, J.; Laxalde, D.; Perktold, J.; Cimrman, R.; Henriksen, I.; Quintero, E. A.; Harris, C. R.; Archibald, A. M.; Ribeiro, A. H.; Pedregosa, F.; van Mulbregt, P.; SciPy 1.0 Contributors. SciPy 1.0: Fundamental Algorithms for Scientific in Python. *Nat Methods* **2020**, *17*, 261–272. https://doi.org/10.1038/s41592-019-0686-2.

(9) Hunter, J. D. Matplotlib: A 2D Graphics Environment. *Comput Sci Eng* **2007**, *9* (3), 90–95. https://doi.org/10.1109/MCSE.2007.55.

(10) Waskom, M. L. Seaborn: Statistical Data Visualization. *J Open Source Softw* **2021**, *6* (60), 3021. https://doi.org/10.21105/joss.03021.

(11) McKinney, W.; others. Data Structures for Statistical Computing in Python. In *Proceedings of the 9th Python in Science Conference*; 2010; Vol. 445, pp 51–56.

(12) Vallat, R. Pingouin: Statistics in Python. *J. Open Source Softw.* **2018**, *3* (31), 1026.

(13) Warren, S. C.; Margineanu, A.; Alibhai, D.; Kelly, D. J.; Talbot, C.; Alexandrov, Y.; Munro, I.; Katan, M.; Dunsby, C.; French, P. M. W. Rapid Global Fitting of Large Fluorescence Lifetime Imaging Microscopy Datasets. *PLoS One* **2013**, *8* (8), e70687.

(14) Chung, C. W.; Stephens, A. D.; Konno, T.; Ward, E.; Avezov, E.; Kaminski, C. F.; Hassanali, A. A.; Kaminski Schierle, G. S. Intracellular Aβ42 Aggregation Leads to Cellular Thermogenesis. *J Am Chem Soc* **2022**, *144* (22), 10034–10041. https://doi.org/10.1021/jacs.2c03599.

(15) Gupta, A. K.; Tulsyan, S.; Bharadwaj, M.; Mehrotra, R. Systematic Review on Cytotoxic and Anticancer Potential of N-Substituted Isatins as Novel Class of Compounds Useful in Multidrug-Resistant Cancer Therapy: In Silico and in Vitro Analysis. *Top Curr Chem* **2019**, *377*, 1–21.
