# Supplementary material for: FLIMPA: A Versatile Software for Fluorescence Lifetime Imaging Microscopy Phasor Analysis
Source: Anal Chem. 2025 May 23;97(22):11382–7. doi: 10.1021/acs.analchem.5c00495 (PMC12163867; doi:10.1021/acs.analchem.5c00495)
Supplement: Supplementary file 2 [file ac5c00495_si_002.pdf]

User Manual

FLIMPA: A Versatile Software for Fluorescence Lifetime Imaging Microscopy Phasor Analysis

Sofia Kapsiani[1], Nino F. Läubli[1], Edward N. Ward[1], Mona Shehata[2], Clemens F. Kaminski[1], Gabriele S. Kaminski Schierle[1]

[1]Department of Chemical Engineering and Biotechnology, University of Cambridge, Cambridge, CB3 0AS, UK
[2]Analytical Sciences, Bioassay, Biosafety and Impurities, BioPharmaceutical Development, AstraZeneca, Cambridge, UK

* Corresponding author: gsk20@cam.ac.uk

Contents

1. Getting started ... 2
2. Loading data ... 2
   2.1. Loading .tif and .tiff files ... 3
   2.2. Loading .ptu files ... 3
   2.3. Loading data alongside manual masks ... 4
   2.4. Loading reference files ... 4
3. Running the phasor plot analysis ... 5
4. Results overview ... 6
   4.1. Fluorescence "Lifetime values" table ... 6
   4.3. Gallery (tau) tab ... 8
   4.4. Gallery (I) tab ... 9
   4.5. Violin plots ... 9
5. Saving FLIMPA's outputs ... 10

User Manual

1. Getting started

- The .exe file for running on Windows can be downloaded using the following link (https://github.com/SofiaKapsiani/FLIMPA/releases)
- Alternatively, FLIMPA can be run by cloning or downloading its GitHub repository as a ZIP file (https://github.com/SofiaKapsiani/FLIMPA) following the instructions provided there.
- Sample Becker & Hickl .sdt files alongside their .tif masks are available on the project's GitHub repository.

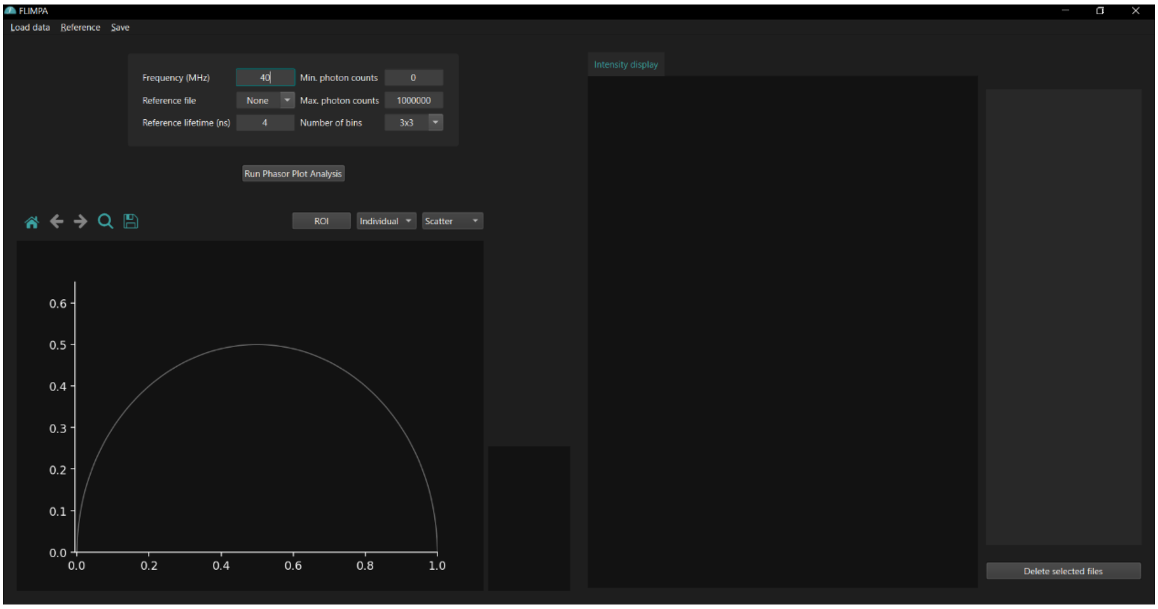

Figure 1. FLIMPA GUI upon launching the software.

2. Loading data

Raw data file formats currently supported:

- **.sdt, .ptu, .tif**

While loading the data, users can choose from the options:

- Define the **experimental condition** for the raw data (e.g., "control"), allowing images with the same condition to be visualised together later and/or
- Provide **manual masks**, in a .tif format, see section 2.3. Loading data alongside manual masks for more information.
- Please note the experimental condition can also be edited after data loading.
- For better gallery visualisation options, files with equal x- and y-dimensions are recommended.

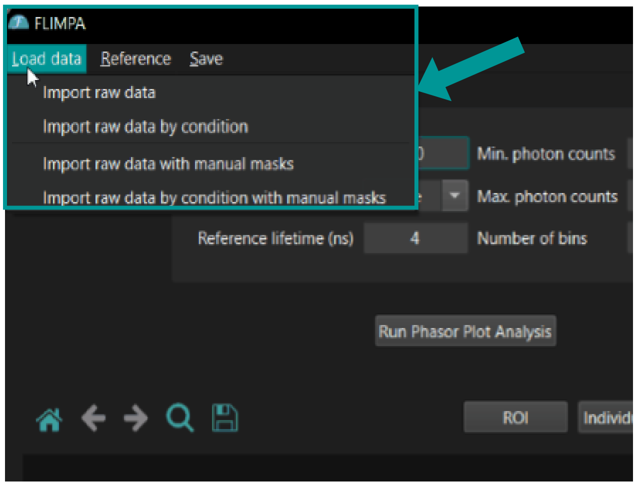

Figure 2. Load data by clicking the "Load Data" button located in the top left corner of the GUI.

2.1. Loading .tif and .tiff files

- Please ensure that your .tif data is in the format (time, x, y).
- When loading your data, users will be prompted to enter the **bin width** (in ns), which is essential for accurate analysis as the file format does not contain temporal metadata.
- If the exact bin width is unknown, FLIMPA provides an estimate option, with the bin width calculated as:

$$\frac{1}{Frequency\ (Hz) \times Number\ of\ bins} \times 10^9$$

- However, this estimate may be inaccurate depending on your data acquisition settings. For the most accurate results, it is recommended to use the actual bin width provided by your instrument.

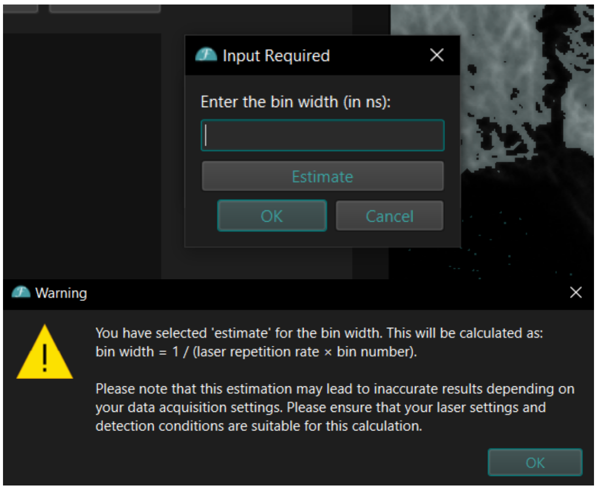

Figure 3. Upon loading .tif or .tiff user will be requested to provide the bin number (in ns). If this is unknown an "estimate" option is available but can lead to inaccurate results.

2.2. Loading .ptu files

- Analysing .ptu files often takes longer than other formats, as these files tend to be larger in both the time and spatial dimensions.
- If your time dimension exceeds 512, we recommend **binning** the **time dimension** to reduce the risk of Python running into memory issues.
- Binning the time dimension will also make the analysis faster. However, if the data have very low photon counts, i.e. fewer than 100 photons per pixel, excessive binning may reduce the accuracy of the results.

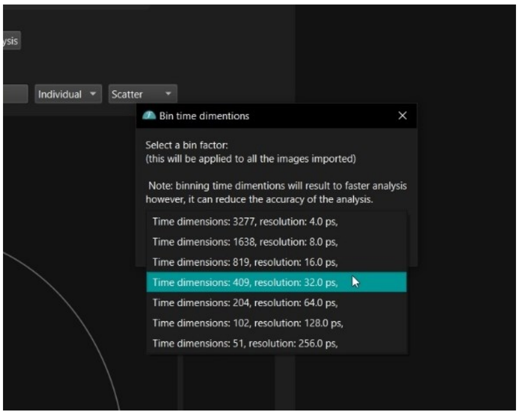

Figure 4. Example time binning options for large .ptu file

2.3. Loading data alongside manual masks

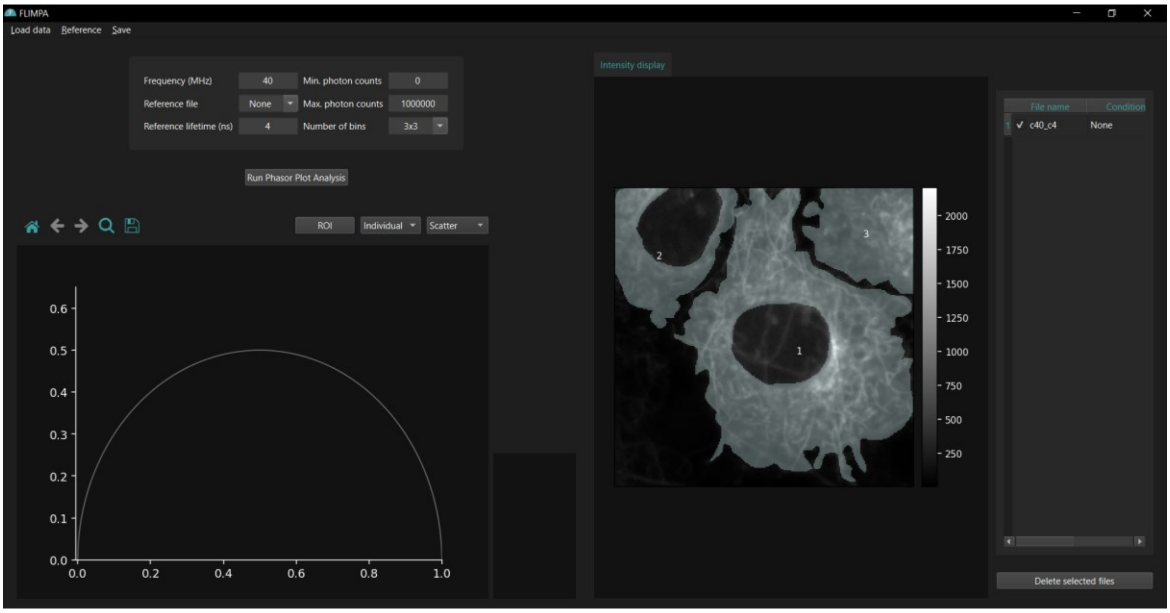

Figure 5. Raw FLIM data can be loaded with their respective manual masks. If the manual masks consist of more than one region of interest (ROI), these will be numbered accordingly.

- Manual masks can be created using FLIMFit and should be named according to the raw data, followed by “segmentation.tif" (e.g., “control.sdt” should have a mask named “control segmentation.tif”).
- Click on “Load data” on the left corner of the GUI, followed by “Import data with manual masks”.
- Select the raw data, then choose the folder containing the masks.
- Once loaded, different ROIs (e.g., cells) will be numbered for separate analysis.
- Alternatively, users can select “Import raw data by condition with manual masks” to assign both the experimental condition and provide the corresponding masks.

2.4. Loading reference files

As a reference file, a **mono-exponential dye** with a **known lifetime** (e.g., Rhodamine 6G with a reference lifetime of 4 ns) must be provided. The file should have equal x- and y- dimensions.

Using an IRF for reference correction

- While measurement of a mono-exponential dye is recommended as a reference file, FLIMPA also accepts **IRF files** in either .sdt or .csv format.
- **Please note that predictions using IRFs are less accurate than using mono-exponential reference samples.**
- The .csv files should contain two columns: the first column representing the time axis at which the IRF was recorded in seconds, and the second column containing the corresponding intensity values.
- The full IRF should be provided without any cropping of the time axis.
- **No other column titles** or any other columns should be included.

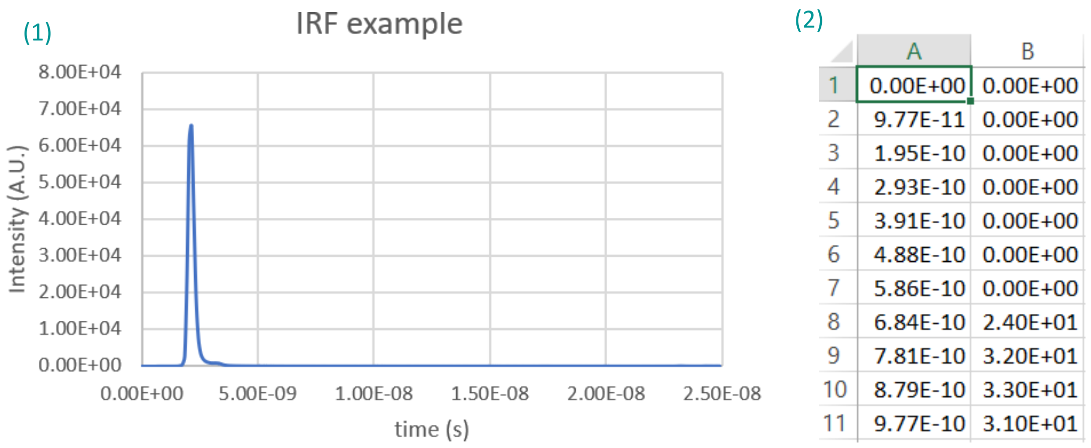

	A	B
1	0.00E+00	0.00E+00
2	9.77E-11	0.00E+00
3	1.95E-10	0.00E+00
4	2.93E-10	0.00E+00
5	3.91E-10	0.00E+00
6	4.88E-10	0.00E+00
7	5.86E-10	0.00E+00
8	6.84E-10	2.40E+01
9	7.81E-10	3.20E+01
10	8.79E-10	3.30E+01
11	9.77E-10	3.10E+01

Figure 6. User guidance for providing .csv IRF files. (1) Example of an IRF with x-axis representing time in seconds (s), while y-axis is the fluorescence intensity. (2) Expected format of the .csv file, with the first column representing the time axis in ns and the second column being the corresponding intensity values. Please note no column titles are provided.

3. Running the phasor plot analysis

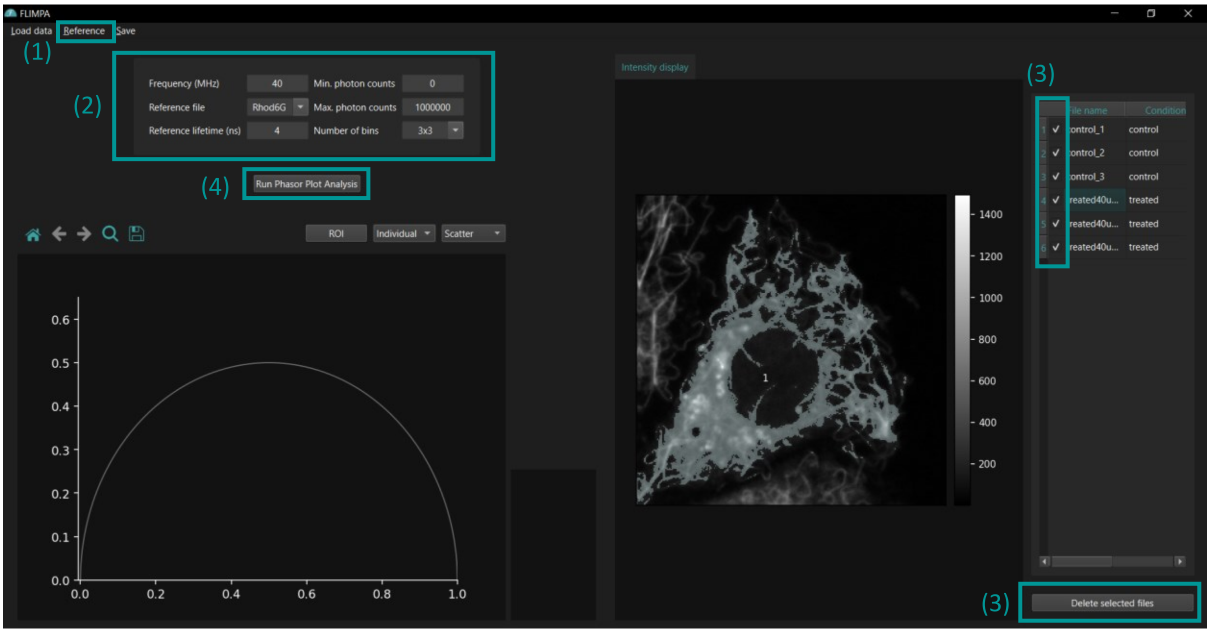

Figure 7. Overview of steps required for running the phasor plot analysis.

1) Load a reference file and set reference lifetime.
2) To run the phasor plot analysis, users will need to specify the following parameters:
    - Laser Frequency (in MHz)
    - Reference File Lifetime (in ns)
    - Number of Time Bins (select from None, 3x3, 7x7, or 9x9)
    - Minimum Photon Count Threshold (optional, at least 100 p.c. per pixel recommended)
    - Maximum Photon Count Threshold (optional)
3) The check box has a dual function:

- Only selected files will be analysed
- Users can click on “Delete selected files” to remove files not needed

4) Click to run the phasor plot analysis.

4. Results overview

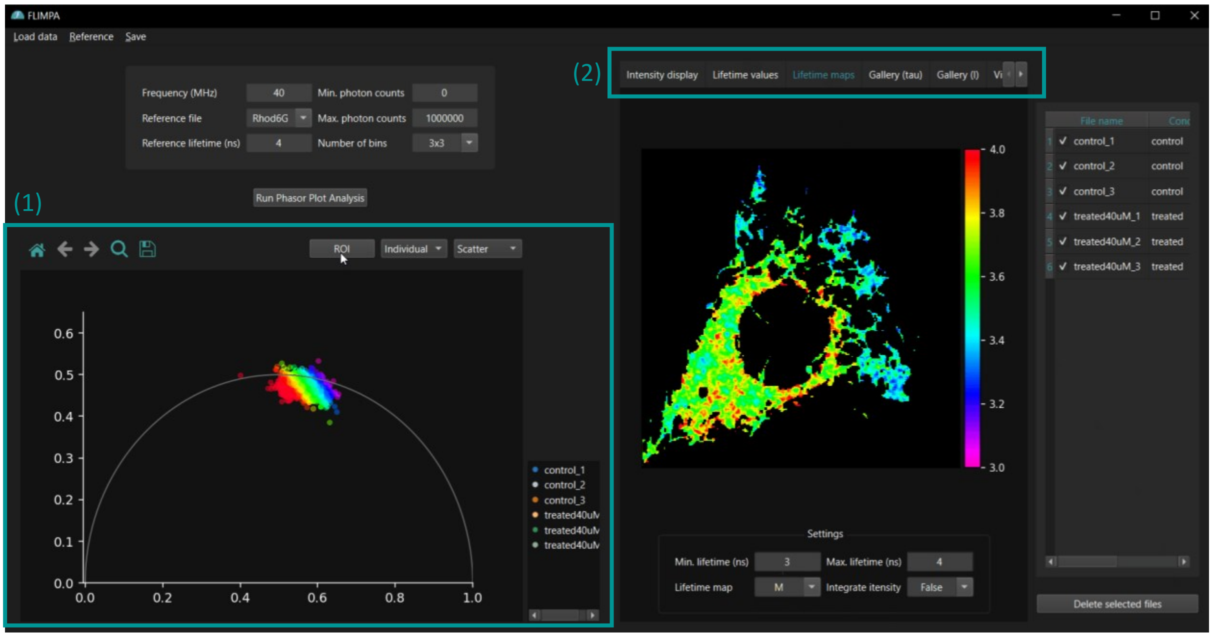

Figure 8. Upon completion of the phasor plot analysis, section (1) will display the phasor plot visualisations, while area (2) will present newly generated tabs containing FLIMPA’s analysis results.

The phasor plot analysis results are displayed across different tabs, specifically the “Lifetime values” table, “Lifetime maps”, “Gallery (tau)”, “Gallery (I)”, and “Violin plots” tabs.

4.1. Fluorescence “Lifetime values” table

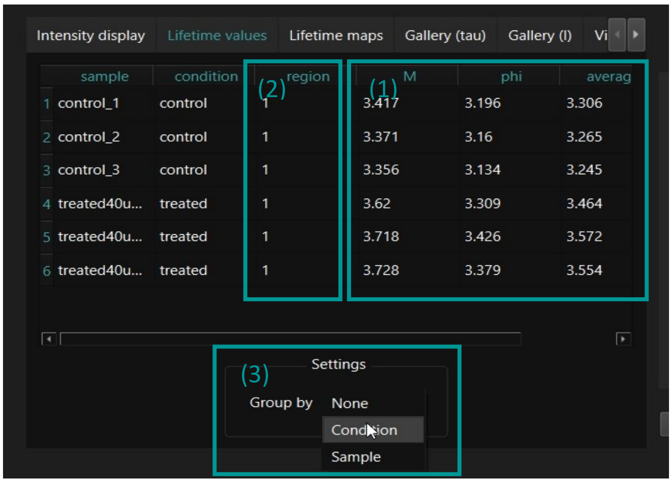

Figure 9. Tab showing the mean fluorescence values per image or ROI in a table format.

1) The table will display the modulation (M), phase (phi), and the average of the modulation and phase fluorescence lifetimes for each image.
2) If different ROIs were provided using manual masks (as shown in section 2.3. Loading data alongside manual masks), the mean fluorescence lifetimes will be listed per ROI instead of per image.
3) Additionally, users can select “Group by Condition” to display the mean lifetime per experimental condition or “Group by Sample” to calculate the mean lifetime per image if different ROIs per image have been provided.

4.2. Fluorescence “Lifetime maps”

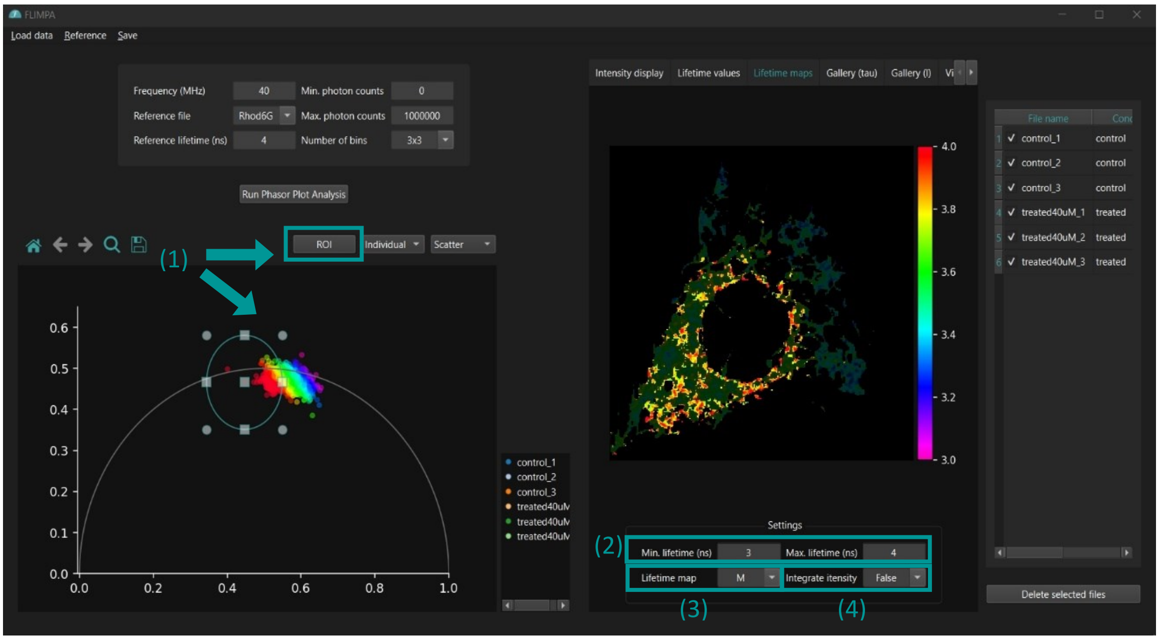

Figure 10. “Lifetime maps” tab displays the individual images where localised effects can be explored using the ROI selection tool.

1) ROI selection tool can be used to investigate localised effects within individual images.
2) Users can set the minimum and maximum lifetime range (in ns) for the fluorescence lifetime map colour bar.
3) Users can select between visualising the modulation (M), phase (phi) or average of modulation and phase lifetime map.
4) If “integrate intensity” is set to “True”, the fluorescence lifetime map will be integrated with the intensity image.

4.3. Gallery (tau) tab

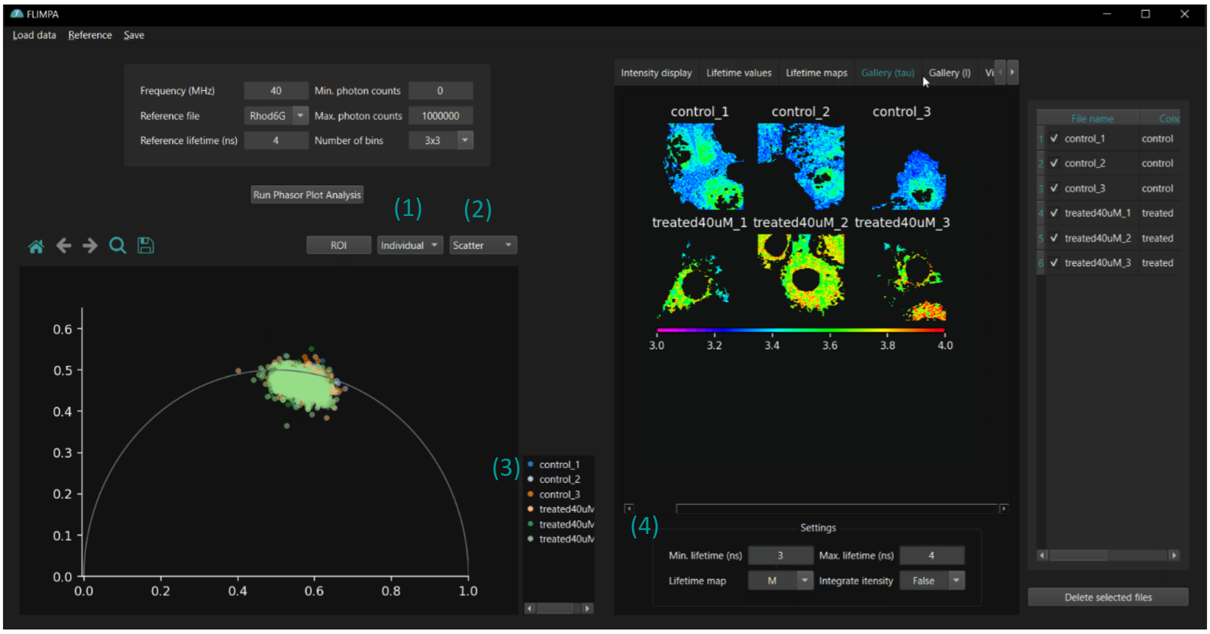

Figure 11. Overview of the "Gallery (tau)" tab, which contains the gallery of the fluorescence lifetime maps of the different samples analysed. The phasor clouds of the samples are displayed in a single plot and can be coloured based on their sample identities (as shown in this Figure) or based on their experimental condition.

1) Phasor clouds can be coloured based on their sample identity (select "Individual") or by their experimental condition (select "Condition").
2) Phasor visualisation options are scatter plots, histograms or contour maps.
3) Legend to highlight entry selected.
4) The gallery of lifetime maps has the same user setting options as the individual lifetime maps shown in Figure 10.

4.4. Gallery (I) tab

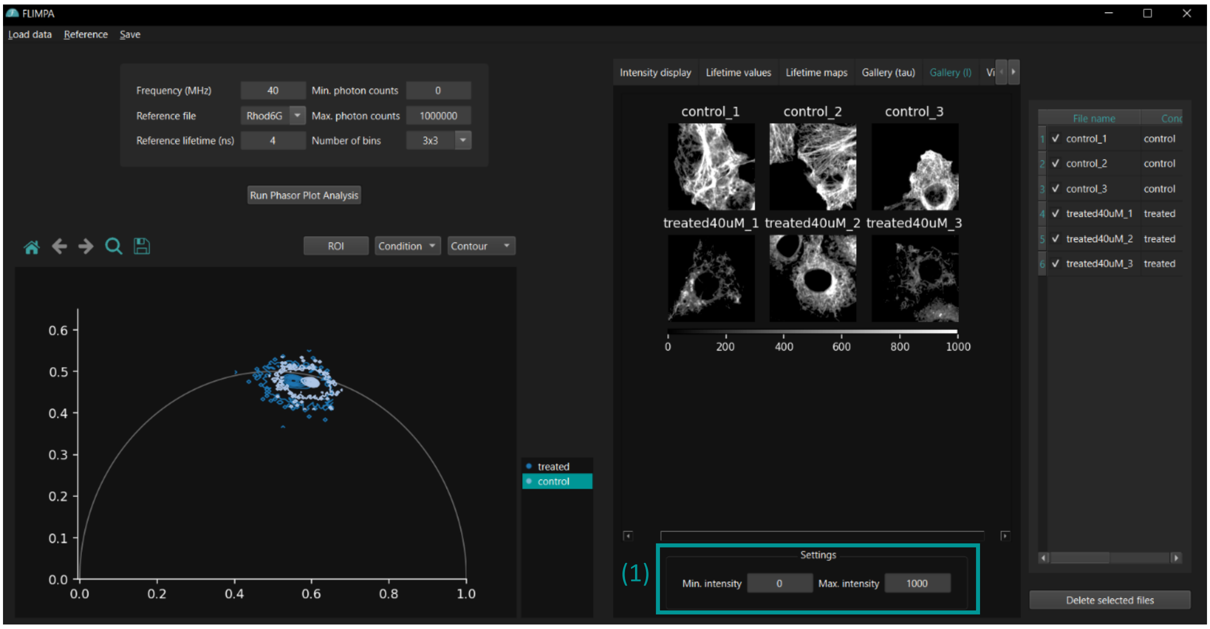

Figure 12. “Gallery (I)” tab provided a gallery of fluorescence intensity maps.

1) Users can set minimum and maximum photon counts per pixel for the fluorescence intensity image colour bar.

4.5. Violin plots

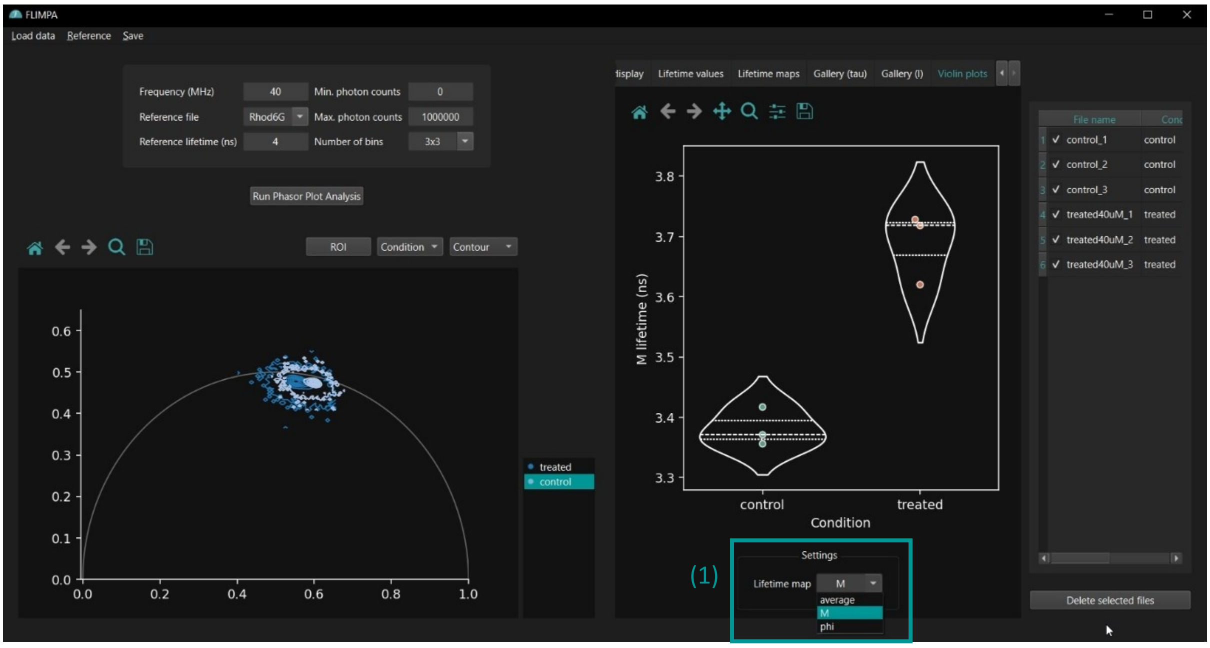

Figure 13. The “Violin plots” tab displays the distribution of data points for each treatment group.

1) Users can select between plotting the mean average, modulation (M) or phase (phi) lifetime per image, or per ROI if manual masks with multiple ROIs have been provided.

5. Saving FLIMPA's outputs

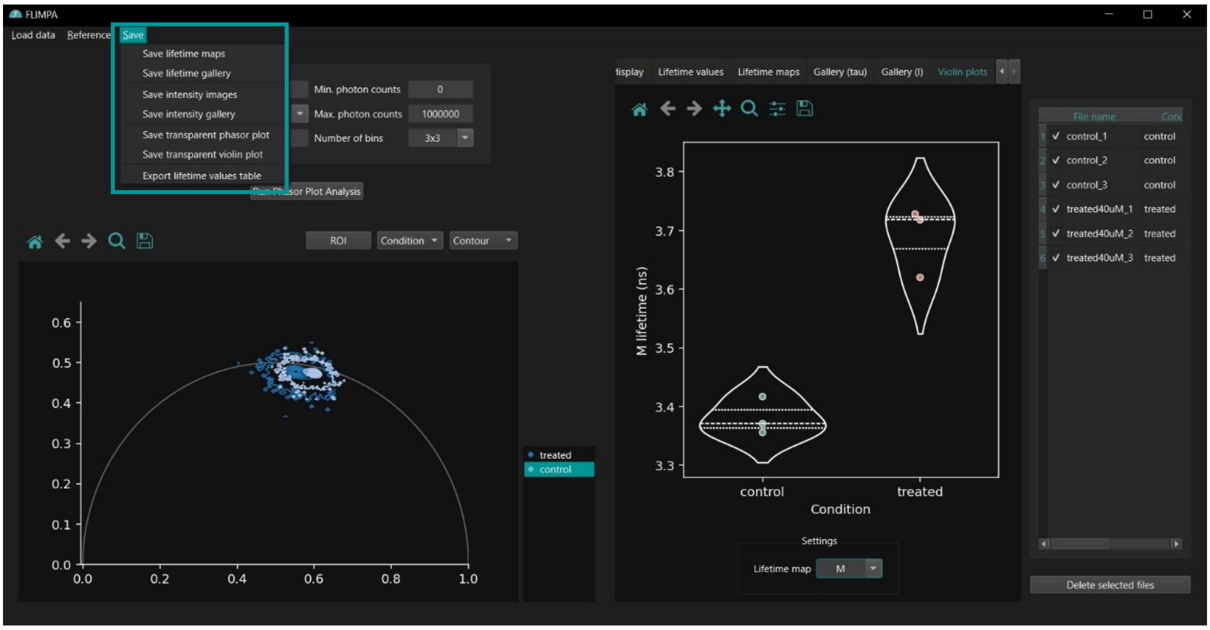

Figure 14. The data generated can be saved by clicking on the "Save" button on FLIMPA's toolbar.

FLIMPA allows users to export all generated data. This includes:

- Lifetime and Intensity Maps: Exported as .png and raw .tif files.
- Gallery Visualisations: Lifetime and intensity galleries exported as .png files.
- Phasor Plots and Violin Plots: Saved with a transparent background.
- Statistical Data: A .csv file containing the mean fluorescence lifetime per image can be exported for further statistical analysis.
